# *Caryopteris odorata* and its metabolite coumarin attenuate characteristic features of cardiometabolic syndrome in high-refined carbohydrate-high fat-cholesterol-loaded feed-fed diet rats

**DOI:** 10.3389/fphar.2023.1097407

**Published:** 2023-03-23

**Authors:** Mobeen Ghulam Ahmed, Malik Hassan Mehmood, Shumaila Mehdi, Maryam Farrukh

**Affiliations:** Department of Pharmacology, Faculty of Pharmaceutical Sciences, Government College University of Faisalabad, Faisalabad, Pakistan

**Keywords:** *Caryopteris odorata*, leptin, adiponectin, chemerin, diet induced cardiometabolic syndrome

## Abstract

*Caryopteris odorata* (D. Don) B.L. Robinson (Verbenaceae family) is an aromaric shrub traditionally used to treat diabetes and related pathologies (diabetic foot ulcer), cancer/tumors, wound healing, and inflammation. It is enriched with flavonoids and phenolics like coumarins, quercetin, gallic acid, coumaric acid, stigmasterol, α-tocopherol, and iridoids. *C. odorata* has been reported as having α-glucosidase, anti-inflammatory, and anti-oxidant properties. Its effectiveness in preventing cardiometabolic syndrome has not yet been assessed. This study aims to investigate the potential efficacy of *C. odorata* and coumarin for characteristic features of cardiometabolic syndrome (CMS), including obesity, dyslipidemia, hyperglycemia, insulin resistance, and hypertension by using high-refined carbohydrate-high fat-cholesterol (HRCHFC)-loaded feed-fed rats. Chronic administration of *C. odorata* and coumarin for 6 weeks revealed a marked attenuation in body and organ weights, with a consistent decline in feed intake compared to HRCHFC diet fed rats. The test materials also caused a significant reduction in the blood pressure (systolic, diastolic, and mean) and heart rate of HRCHFC-diet fed rats. Improved glucose tolerance and insulin sensitivity tests were also observed in test material administered rats compare to only HRCHFC-diet fed rats. *C. odorata* and coumarin-treated animals produced a marked decline in serum FBG, TC, TG, LFTs, and RFTs, while an increase in serum HDL-C levels was noticed. *C. odorata* and coumarin also significantly modulated inflammatory biomarkers (TNFα, IL-6), adipokines (leptin, adiponectin, and chemerin), and HMG-CoA reductase levels, indicating prominent anti-inflammatory, cholesterol-lowering, and anti-hyperglycemic potential. Administration of *C. odorata* and coumarin exhibited a marked improvement in oxidative stress markers (CAT, SOD, and MDA). Histopathological analysis of liver, heart, kidney, pancreas, aorta, and fat tissues showed a revival of normal tissue architecture in *C. odorata* and coumarin-treated rats compared to only HRCHFC-diet fed rats. These results suggest that *C. odorata* and coumarin possess beneficial effects against the characteristic features of CMS (obesity, insulin resistance, hypertension, and dyslipidemia) in HRCHFC feed-administered rats. These effects were possibly mediated through improved adipokines, glucose tolerance, and insulin sensitivity, the attenuation of HMG-CoA reductase and inflammatory biomarkers, and modulated oxidative stress biomarkers. This study thus demonstrates a rationale for the therapeutic potential of *C. odorata* and coumarin in CMS.

## Introduction

Cardiometabolic syndrome (CMS) is a consolidation of metabolic anomalies characterized by central obesity, insulin resistance, hyperglycemia, hyperlipidemia, and hypertension (Ojetola et al., 2021). It is reaching pandemic levels and hence presents a serious global health concern (Agrawal et al., 2018). CMS prevalence varies and depends greatly upon population, gender, age, and race. It is directly proportional to the increased frequency of obesity, which is considered an underlying cause of CMS (Ojetola et al., 2021). It affects approximately 25% of the world’s adult population. According to a survey conducted by the Dow University of Health Sciences, Karachi, Pakistan, ageing and unhealthy lifestyle practices have led to an upsurge in CMS risk factors which affect more than 51.8% adults—of these, 39.7% had hypertension, 29.7% had obesity, 23.1% diabetes, and 11.9% had dyslipidemia (Inam and Shah, 2019). Increased oxidative stress and inflammation are the key factors in obesity which lead to a sequential progression in insulin resistance (IR), type 2 diabetes, dyslipidemia, hepatic steatosis, and hypertension (Sharma et al., 2012), resulting in the development of CMS. Metabolic syndrome and chronic inflammation have an established association with increased adipocytokine (TNFα, IL-6, leptin, and chemerin) and decreased adiponectin levels (Nwakiban-Atchan et al., 2022).

Dietary habits and lifestyle modifications are known to ameliorate the risk factors of CMS for a limited duration. Similarly, weight reduction also attenuates cardiometabolic abnormalities; however, these modifications remain unsuccessful when practiced intermittently in established CMS. Pharmacological treatment involves the use of multiple therapeutic agents, but with increased cost, patient non-compliance, and adverse effects (Gutierrez-Salmean et al., 2014). Hence, there is an urgent need to develop multifactorial approaches to the treatment of CMS to probe its underlying causes. Due to the high prevalence of CMS, WHO recommends an increased use of medicinal herbs/plants to combat its characteristic features. Multiple evidence also indicates that medicinal plants and naturally derived compounds have great potential to combat CMS and related pathologies (Agrawal et al., 2018; De-Oliveira et al., 2021; Ojetola et al., 2021).

*Caryopteris odorata* (D. Don) B.L. Robinson, belonging to family Verbenaceae, mostly exists in tropical and subtropical climatic regions. The Verbenaceae family comprises of numerous important medicinal plants with a wide range of biological activities and potent phytochemical constituents. In Pakistan, around 35 species and 17 genera of this plant have been found (Shahzadi et al., 2013). It is a shrub which is found mostly in the subtropics or outer Himalayas of Pakistan, Bhutan, India, and Bangladesh. In Pakistan’s traditional system of medicine, its powdered leaves and flowers are used to treat diabetes and associated pathologies like diabetic foot ulcer, tumors/cancer, wound healing, and general body aches (Abbasi et al., 2017; Joshi et al., 2019). It is used as fuel and fodder in northern areas of Pakistan. It has also been claimed to have antiulcer, antitumor, antidiarrhea, anti-inflammatory, antirheumatic, antihemorrhagic, and antitussive effects in Pakistan, China, and Mongolia (Frezza et al., 2019). It has been used for allergic reactions in Nepal (Bhattarai and Tamang, 2017).

The pharmacological effects of *C. odorata*, its derived iridoids, and the essential oils of its leaf, stem, and flower feature superodixe dismutase (SOD) and nitric oxide (NO) scavenging (Joshi et al., 2019), and antioxidant (Shahzadi et al., 2011; Shahzadi et al., 2012; Shahzadi et al., 2013; Abbasi M. A. et al., 2014; Singh et al., 2014; Joshi et al., 2019) and lipid peroxidation inhibitory activities (Shahzadi et al., 2011). It has also been reported to possess anti-inflammatory (Joshi et al., 2019), antimicrobial, antifungal, hemolytic (Abbasi M. et al., 2014; Singh et al., 2014), anti-urease, and anti-tyrosinase properties (Shahzadi et al., 2012). Furthermore, *C. odorata* has also shown enzyme inhibition potential against α-glucosidase, acetylcholinesterase, butylcholinestrase, and lipo-oxygenase (Shahzadi et al., 2013; Abbasi M. et al., 2014), thus providing indirect evidence for its use in diabetes, obesity, dyslipidemia, and hypertension. *C. odorata* has also been reported to be enriched with diverse phytoconstituents, including quercetin, gallic acid, cinnamic acid, vanillic acid, coumaric acid, ursolic acid, coumarins, and their derivative furanocoumarin (psoralen, methoxsalen, oxypeucedanin, isoimperatorin, and bergamottin) (Abbasi et al., 2017), stigmasterol, α-tocopherol, β-caryophyllene, α-and β-longipinene, α-humulene, caryophyllene oxide, germacrene B and D, and α-bisabolol. Various iridoid glycosides (8-O-trans-cinnamoyl caryoptoside, 8-O-trans-cinnamoyl shanzhiside methyl ester, 8-O-trans-cinnamoyl mussaenoside, 8-O-caffeoyl massenoside) have also been isolated from *C. odorata.* These possess widespread cardiovascular protective, hepatoprotective, cholagogues, antihyperglycemic, analgesic, anti-inflammatory, anti-cancer, spasmolytic, antitumor, antiviral, immune modulator, and laxative (Shahzadi et al., 2013; Joshi et al., 2019) properties, thus providing support for the use of *C. odorata* in treating CMS. Despite the available precious pharmacological profile of *C. odorata,* no study has explored its pharmacological effects on CMS.

Coumarins owe their class name from *coumarou*, the vernacular name of the tonka bean (*Dipteryx odorata*). Coumarins were first isolated from the tonka bean by Vogel in 1820 (Bruneton, 1999). They are also referred as “plant-derived secondary metabolites”. Coumarins (1,2-benzopyrone or o-hydroxycinamic acid-8-lactone) constitute a large class of phenolic substances; they are abundantly present in many plants like woodruff, tonka beans, cinnamon, green tea, honey, fruits (cloudberry, bilberry), celery, carrots, and chicory (Lake, 1999). Coumarin possesses anti-oxidant properties, which is evident from its ability to protect cells from oxidative damage and stress (Basile et al., 2009). It has also shown anti-inflammatory properties through stimulation of phagocytosis, enzyme production, and proteolysis to removes protein and edematous fluid from injured tissue sites (Piller, 1975). Coumarin inhibits COX and LOX enzymes, produces SOD (Fylaktakidou et al., 2004), and inhibits protein expression of NO synthase and COX-2 enzyme (Huang et al., 2012). It possesses promising therapeutic potential as an anticoagulant (Abdelhafez et al., 2010) and antithrombotic (Jain et al., 2013) agent for cardiovascular disorders. It has also been considered a vitamin K antagonist because of its potential to interfere with vitamin K cycle conversion (Hirsh et al., 2001). It displays an antihypertensive effect (Nguelefack-Mbuyo et al., 2008) through its smooth muscle relaxant actions (Mead et al., 1958). Coumarin is also known for its effectiveness in reducing left ventricular hypertrophy (Najmanova et al., 2015); it has peripheral and coronary vasodilatory efficacy (Gantimur et al., 1986) through platelet aggregation and NO-mediated system and has thus also been used to treat angina pectoris (Najmanova et al., 2015). Coumarin has also shown an antihyperglycemic effect (Murali et al., 2013). It has exhibited antihyperlipidemic action—possibly by activating the AMPK phosphorylation pathway and downregulating FAS and HMGR protein expression (Iwase et al., 2017; Li et al., 2017). Coumarin also possesses antiadipogenic effects. It is known to inhibit lipid accumulation and lipogenic-related gene expressions in 3T3-L1 adipocytes cells, possibly through the PPARγ pathway (Shin E. et al., 2010; Taira et al., 2017). Although coumarin has been studied in isolation and, in part, for its pharmacological effect in hypertension, diabetes, and dyslipidemia, no study has comprehensively ascertained its pharmacological effects against characteristic features of CMS using a rat model.

This study has been designed to provide a pharmacological basis for the protective potential of *C. odorata* and coumarin in CMS, including obesity, hyperglycemia, insulin resistance, hypertension, and dyslipidemia using HRCHFC-loaded diet-fed rats. This study also explains the possible mechanism(s) of *C. odorata* and coumarin for anti-oxidant, anti-hypertensive, anti-inflammatory, and HMG-CoA reductase inhibitory pathways. It thus presents sound evidence for the use of *C. odorata* in CMS. Furthermore, anti-obesity and insulin sensitizing effects have also been endorsed by the modulating potential of *C. odorata* and coumarin on adipokinin levels (leptin, adiponectin, and chemerin), and inflammatory (TNFα, IL-6) and oxidative stress biomarkers (CAT, SOD, MDA).

### Novelty

• This is a pioneer study to show the effectiveness of *C. odorata* and coumarin in CMS induced by a HRCHFC-loaded diet.
• The quantification of obesity-related candid parameters including chemerin, leptin, adiponectin, and the inhibition of HMG-CoA reductase for the efficacy of *C. odorata* and coumarin in CMS.
• *C. odorata* and coumarin exhibit anti-inflammatory and antioxidant potential, which might reflect their effectiveness in CMS.

## Materials and methods

### Chemicals and drug

Coumarin ≥99% (HPLC), metformin, rosuvastatin, formaldehyde, and cholic acid were purchased from Sigma Aldrich (St. Louis, MA, United States). Cholesterol was bought from PanReac AppliChem (Ottoweg, Darmstadt, Germany). Additional ingredients, including sodium chloride (NaCl), dry powdered milk (Nido/Everyday, Nestle Ltd, Lahore, Pakistan), vegetable oil (Dalda oil, Unilever, Lahore, Pakistan), desi ghee (Pak pure, United Dairy Pharms, Lahore, Pakistan), and multivitamins (Metavit-Super, Batch# PM5483, Prix Pharmaceuticals (Pvt.) Ltd. Lahore, Pakistan), were purchased from respective commercial suppliers. Choker, highly refined wheat flour, fishmeal, molasses, and potassium metabisulfite were obtained from the local market in Faisalabad, Pakistan. All drug solutions and dilutions of the aqueous methanolic extract of *C. odorata* were prepared freshly on daily basis in distilled water. Stock solution of coumarin was prepared in 1% (v/v) dimethyl sulfoxide (DMSO) and 1% Tween-80 (w/v). All the chemicals and drugs used in this study were of analytical grade purity.

### Plant collection, identification and preparation of extract

Whole plant material was obtained from Poonch, Azad Jammu Kashmir (AJK), Pakistan, in May 2020. Dr. Sardar Irfan Mehmood, Department of Botany, Govt. Boy’s Degree College Abbasapur, Poonch AJK, Pakistan, identified and authenticated the plant material. The crude specimen was also submitted to the AJK Medicinal Plants Herbarium (AJKMPH) with issued Voucher no. AJKH: 437 for future reference. Whole plant of *C. odorata* was shade dried and ground into a fine powder in a mechanical grinder. Approximately 1.5 kg of fine ground powder was soaked in methanol and distilled water (80:20) for 7 days at 25°C. The first filtrate was collected by passing soaked solution through muslin cloth followed by Whatman filter paper No.1. The maceration process was repeated thrice to obtain a sufficient quantity of extract. A rotary evaporator (Model: RE300 Stuart ® United Kingdom) was used to evaporate all of the filtrates. After air drying the final filtrate, the yield of C. *odorata* aqueous methanolic extract was 9% wt/wt.

### Animals

Wistar albino rats weighing 180–220 g were used in this experiment. These were housed in an animal house at standard conditions of 12 h light and dark cycle, 55% relative humidity, and 22 ± 3°C temperature with free access to food and water. All the experiments were performed according to standard housing conditions and laboratory animal protocols as approved by the animal ethical review committee of GCU, Faisalabad No. IRB: 879 (Ref. No. GCUF/ERC/2279).

### Quantitative analysis of *C. odorata*

#### Estimation of total flavonoid contents (TFC)

*C. odorata* extract (500 µL) was mixed with 2 ml of distilled water and 0.15 ml of NaNo_2_ solution (5%). Next, 150 µL of 10% aluminum chloride (AlCl_3_) solution with 4% sodium hydroxide solution were added into it. Total volume was made up to 5 ml with methanol. After incubation for 15 min, absorbance was measured at 510 nm. The findings were displayed as (mg/g QE) of catechin equivalent of the concentration of plant extract (Kumar and Roy, 2018).

#### Estimation of total phenolic contents (TPC)

*C. odorata* extract was prepared at 0.1 g/ml concentration, and 200 µL (two replicate) was poured in a test tube. Folin Ciocalteu reagent (6%) 1 ml and 0.8 ml of sodium carbonate (Na_2_CO_3_) solution (7.5% or 0.6 M) were added to the mixture. The test tube mixture was thoroughly mixed and incubated for 30 min. Absorbance was recorded at a wavelength of 765 nm or 725 nm. Total phenolic contents (TPC) were expressed as mg of gallic acid equivalent in milligram GAE/g of dry plant extract. Gallic acid was used as a reference control (Fetni et al., 2020).

#### DPPH (1,1-diphenyl-2picryl-hydrazyl) radical scavenging activity

The anti-oxidant potential of *C. odorata* extract was accessed using DPPH by following an earlier practiced method with slight modification (Fetni et al., 2020). To prepare fresh stock solution, 4 mg of DPPH was mixed with 100 ml methanol. A 3 ml aliquot was prepared with 2,800 µL of DPPH and 200 µL from different concentrations of *C. odorata* extract (500–6.25 μg/ml). This mixture was stored for 30 min in the dark at 25°C. OD (optical density) was noted at 517 nm. Methanol and DPPH were used as negative controls while, methanol was used as blank control. The antioxidant activity of quercetin (standard) was also evaluated. The % age inhibition of the DPPH radical scavenging capacity of the test samples was computed as: scavenging activity/DPPH % age inhibition = absorbance of negative control − absorbance of test sample/absorbance of negative control × 100.

#### Estimation of reducing power

A 10 µL test sample and 25 µL each of 1% potassium ferrocyanate (K₄ [Fe (CN)₆] ·3H₂O), 0.2 mM (pH = 6.6) phosphate buffer, and (10%) trichloroacetic acid were added to the test tube. It was then centrifuged to separate the supinated layer. Then, 8.5 µL of ferric chloride and 85 µL of distilled water were added. The mixture was incubated for 30 min and absorbance was recorded at a wavelength of 700 nm. The ferric reducing power of the sample was evaluated using following formula:

% age reducing power = A_o_-At/A_o_ × 100 where A_o_ = negative control absorbance, At = test sample absorbance (Sharma et al., 2016).

### Determination of H_2_O_2_ scavenging activity

Hydrogen peroxide (H_2_O_2_) (40 mM) solution was freshly prepared in 50 mM phosphate buffer (PBS) (pH = 7.4). Different concentrations of *C. odorata* extract and standard 10–200 μg/ml (50–100 µL) were added to the H_2_O_2_ solution (0.6 ml). After 10 min of incubation, the change in absorbance was measured from 30 s to 3 min at a wavelength of 230 nm. The % age of H_2_O_2_ scavenged by the sample was calculated using following formula:

% age of H_2_O_2_ scavenging activity = A_o_ -At/A_o_ × 100, where A_o_ = negative control absorbance, At = test sample absorbance (Sharma et al., 2016).

### Finger print analysis by Fourier transform infrared (FTIR) spectroscopy

An ATR-FTIR spectrophotometer (Alpha-Bruker, Germany) was used for FTIR analysis of *C. odorata* extract. The sample was supplied with the projected ATR (Attenuated Total Reflectance) accessory system. ATR potassium bromide diamond crystal was thoroughly cleaned with alcohol during procedure. Around 10 mg of *C. odorata* extract was prepared and cautiously placed on the surface of the FTIR diamond crystal center. The spectrum was analyzed at an absorbance range of 400–4,000 cm^–1^ with a resolution of 4 cm^–1^ (Kumar and Roy, 2018).

### High performance liquid chromatographic (HPLC) analysis

The quantifiable determination of flavonoids and phenolics present in *C. odorata* was conducted using the HPLC technique. A sample of *C. odorata* extract (50 mg) was prepared with methanol (24 ml), distilled water (16 ml), and hydrochloric acid (10 ml, 6 M). It was then incubated at 95°C for 2 h. The prepared solution was purified by membrane filter paper (nylon, 0.45 μm). A gradient reverse phase HPLC system (Shimadzu, Japan Model = SPD-10AV) was utilized to isolate the phenolics and flavonoids from *C. odorata*. The system was provided with C_18_ (shim-pack-CLC-ODS) 5 μm column (25 cm × 4.6 mm) attached with an UV-visible spectrophotometer detector system and automatic injector for sampling. Separation of the valuable constituents was achieved through a gradient mobile phase (A: acetic acid and water; B: acetonitrile, 1 ml/min flow rate). Solvent B was used at a different gradient from time to time, like 15% for 0–15 min, 45% for 15–30 min, and 100% for 35–45 min. The compounds were detected at a preset wavelength of 280 nm and temperature of 25°C. Results were recorded by comparing (Rt) retention time with UV-visible peaks previously gained from the standard compound(s). Quantification was achieved by external standardization (Fetni et al., 2020).

Similarly, the standardization of coumarin was carried out in the isocratic mode using acetonitrile (40%)/water (60%); v/v. An injection volume of 20 μL with a flow rate of 0.5 ml/min was used at 274 nm UV detection. Quantification of coumarin in the plant sample was performed by an external standard method by comparison with coumarin (Sigma-Aldrich) as a standard. The stock solution of plant sample was prepared by mixing 224 mg of the dry extract in a 50 ml solution of methanol/water (80:20). The HPLC chromatogram was obtained using the same mobile phase and detection wavelength as for coumarin (Celeghini et al., 2001).

### Preventive effect of *C. odorata* and coumarin on HRCHFC diet induced CMS

A high-refined carbohydrate-high fat-cholesterol loaded diet was used to induce CMS in animals (Agrawal et al., 2018).

### Animal diet preparation

Two different types of diets were prepared for the study.

### Standard diet (SD)

SD used the following composition for a 15 kg diet: chokar (5 kg), high-refined wheat flour (5 kg), dried powdered milk (2 kg), molasses (150 g), powdered fishmeal (2.25 kg), sodium chloride (75 g), potassium metabisulfite (15 g), vegetable oil (500 g), desi ghee (1 kg), and multivitamin (33 g). All the solid ingredients were ground and mixed to form palatable biscuits for the animals. Water was added in small quantities to make a soft composition (Aziz et al., 2013).

### High-refined carbohydrate-high fat-cholesterol loaded diet (HRCHFC)

An earlier protocol was followed with minor modifications (Aziz et al., 2013). The HRCHFC diet comprised 20% protein, 35% carbohydrate, and 50% fat combined with cholesterol (2%w/w) and cholic acid (0.5% w/w).

### Experimental design

Wistar albino rats were randomly allocated into eight different groups of six animals prior to dietary manipulation. The animals in Group 1 were considered the negative control and received a normal or standard diet, while the remaining groups, II–VIII, were administered the HRCHFC diet for 12–14 weeks. Group II was considered the disease control, receiving the HRCHFC diet only. The animals in Groups III–VI were further divided into different treatment groups. Group III was given metformin at 300 mg/kg/d, while group IV was administered metformin + rosuvastatin at (200 mg/kg/d + 1.5 mg/kg/d); these were considered the positive controls. Coumarin at 30 and 70 mg/kg was administered to Groups V and VI, serving as treatment. An aqueous methanolic extract of *C. odorata* (150 and 300 mg/kg) was administered to Groups VII and VIII as treatment. The treatment schedule was started orally from the eighth week of HRCHFC diet intake and continued until the study was terminated (Agrawal et al., 2018).

The doses of coumarin were selected from the results of our preliminary pilot experiment performed on few animals at given doses (appreciable effects were observed against developed parameters of CMS; data not shown) and the dose range of coumarin used in previous animal studies (Pari and Rajarajeswari, 2009) and doses exhibiting hepatic toxicity (Tasdemir et al., 2017).

Considering that the traditional dose of *C. odorata* plant used in human is 1 teaspoon thrice daily (Yaseen et al., 2015), the doses of *C. odorata* extract were selected on the basis of converting human doses to animal doses (Shin J. W. et al., 2010), and the effective dose range of similar species used in animal models (Ullah et al., 2019).

### Body weight, feed intake and weight of different body organs

Body weights (g) were recorded at Weeks 0, 2, 4, 6, 8, 10, 12, and 14 to assess weight variation. Feed intake (g) was measured daily. Different organ weights (g) of the animals were calculated at the completion of study, such as the liver, heart, and kidney (Sasso et al., 2019).

### Non-invasive measurement of blood pressure (NIBP) in conscious rats

Blood pressure was assessed by the tail cuff method in conscious rats. The systolic pressure of the conscious rats was recorded non-invasively using a tail cuff attached with a PowerLab data acquisition system (AD Instruments, Sydney, Australia) at an interval of 2 weeks. For estimation of blood pressure, the cuff sensor was attached to their tails by keeping the rat in a NIBP restrainer of appropriate size. The expected pressure (SBP, 200 mm Hg) was applied to the tail cuff by inflation followed by its gradual deflation. The intensified pulses were noted by PowerLab on Lab chart 7.0 running on a computer during inflation and deflation. Various hemodynamic parameters like systolic blood pressure (SBP), mean blood pressure (MBP), and heart rate (HR) were directly monitored by pulse tracing. Diastolic blood pressure (DBP) was measured by the formula DBP= (3MBP-SBP)/2. Blood pressure parameters were monitored at Weeks 0, 4, 8, 10, 12, and 14 of the diet in conscious rats. For each measurement, an average of 3–5 pressure readings was recorded (Senaphan et al., 2015).

### Determination of fasting blood glucose (FBG), oral glucose tolerance test (OGTT), and insulin sensitivity/tolerance test (ITT)

Fasting blood glucose levels (FBG) were measured from overnight-fasted rats using a digital glucometer (EVOCHECK GM700S) at the end of treatment. During the 14th week of treatment, the animals were subjected to OGTT to measure the effect of *C. odorata* and coumarin administration on glucose metabolism. All rats had been fasted for 12 h. Afterwards, a glucose load (2 g/kg, p.o.) was administered to all test animals. Blood was drawn from their tail veins to assess the glucose levels in the test samples at 0, 30, 60, 90, and 120 min after glucose load through a digital glucometer (EVOCHECK GM700S). Insulin tolerance was tested at Day 5 before the animals were euthanized. ITT was conducted after 6 h of food deprivation. Blood glucose levels were verified in the animals at a fed state (0 min). Thence, 0.75 U/kg/animal weight of insulin (100 U/ml) was administered through intraperitoneal injection. Blood samples were drawn to assess glucose levels at 15, 30, 60, and 120 min by digital glucometer (EVOCHECK GM700S). Area under curves (AUC) was determined for each animal to calculate the mean for the whole study group (Sasso et al., 2019).

### Biochemical analysis

After the 14th week of the experiment, the animals were starved for 18 h before being euthanized after deep anesthesia with isoflurane (5–10% v/wt) through inhalation in a closed chamber. Blood was drawn by cardiac puncture from each rat. The collected blood samples were then centrifuged at 4,000 rpm for 10 min to obtain the serum and stored at −80°C. Serum samples were preserved for further biochemical analysis (Javaid et al., 2021).

### Assessment of lipid, liver, and kidney profile indices

The lipid profile including triglycerides (TG), high density lipoprotein (HDL), total cholesterol (TC), liver profile indices (aspartate aminotransferase (AST), alanine aminotransferase (ALT), and renal profile indices (urea and creatinine) were measured from isolated serum samples using commercial biochemical assay kits (Germany) as per the manufacturer’s protocol. The results were shown in mg/dl and U/L (Sasso et al., 2019).

### Determination of leptin, adiponectin, chemerin, and HMG-CoA reductase levels

Serum biomarkers for obesity, hyperlipidemia and insulin resistance, leptin (E-EL-R0582), adiponectin (E-EL-H6122), HMG-CoA reductase (E-EL-H2472) were sourced from Elabscience, United States, and chemerin (E-0864Ra) from BT LAB, Birmingham, United Kingdom; levels were analyzed by ELISA Kits as per manufacturer’s instructions. 100 µL of serum (reaction mixture) in pre-coated wells with specific rat antibodies of LEP, ADP, HMG-CoA reductase, and CHEM, maintained at 37°C in an ELISA plate reader (DIA source, Germany), were taken. Responses were checked at 450 nm wavelength. 10 µL of serum leptin was taken and diluted 50-fold. The dilution factor was multiplied with sample OD. Serum adiponectin and chemerin levels were shown as pg/ml and ng/ml, respectively, while leptin and HMG-CoA reductase levels were expressed as ng/mL and pmol/ml, respectively (Della-Vedova et al., 2016).

### Determination of inflammatory biomarkers (TNF-α and IL-6)

Serum concentration of TNF-α (E-EL-H0109) and IL-6 (E-EL-H0102) were determined using ELISA Kits (Elabscience Biotechnology CO. Ltd., United States) with sensitivity in the 7.6 pg/ml – 46.88 pg/ml range. Assays were performed as per manufacturer's protocol. The reaction mixture was provided 100 μL of serum in previously coated wells in the ELISA plate reader (DIA source, Germany) and kept at 37°C. The reactions were measured at an absorbance of 450 nm. The findings are presented as pg/ml or pg/mg (Adedapo et al., 2020).

### Determination of oxidative stress biomarkers

The anti-oxidant potential of *C. odorata* and coumarin was analyzed by measuring the amount of catalase, superoxide dismutase, and malondialdehyde peroxidase in tissue homogenate. After 14 weeks, animals were starved for 18 h before being euthanized through deep anesthesia with isoflurane (5–10% v/wt) by inhalation in a closed chamber. Organs (liver, heart, kidney, and aorta) were removed and washed with ice-cold normal saline. These were stored at −80°C for further analysis. 100 mg of each organ was homogenized with 5 ml of tris-base/phosphate buffer solution (7.4 pH) and centrifuged at 2000 rpm for 15–20 min at 4°C. The supernatant layer was removed and stored at -80°C for further analysis. For catalase activity (CAT), 50 µL of different organ homogenates were mixed with freshly prepared H_2_O_2_ (30 mM, 1 ml) and 50 mM phosphate buffer (7.0 pH, 1.95 ml). Absorbance was measured at 240 nm. Catalase activity was estimated as units/g tissue. Superoxide dismutase (SOD) was estimated by following an earlier practiced method (Younis et al., 2018) where chromogen intensity was measured at an absorbance of 560 nm against blank. It was equated with the known SOD standard curve as units/mg. Lipid peroxidation/malondialdehyde peroxidase (MDA) levels were evaluated using the methodology of Adedapo et al. (2020).

### Histological examination

The isolated tissues of heart (5 mm), liver, kidney, aorta, pancreas, and fat (5 μm) were dissected and fixed with 17% formalin. Different organ tissue sections were stained with hematoxylin and eosin. A microtome (Leica, Germany) was used to collect thin sections of organs to assess histopathological changes under a light microscope (ACCU-SCOPE 3001-LED Digital Microscope, United States) (Sasso et al., 2019).

### Statistical analysis

The values were presented as mean ± SEM. The significance among results was tested using one-way analysis of variance (ANOVA) followed by Dunnett‘s test, and two-way ANOVA followed by Dunnett‘s test/Bonferroni post doc test and unpaired *t*-test. Non-linear regression analysis was applied to compare concentration response curves. GraphPad Software 8.4.3 (Diego California United States) was used for statistical analysis and calculation to convert data into graphs.

## Results

### TFC and TPC

The equivalent contents of TF and TP were quantified using standard regression lines of catechin and gallic acid, respectively. TFC of *C. odorata* extract were 67.34 mg catechin/g DE and TPC were 86.82 mg GAE/g DE weight.

### Antioxidant activities

**FIGURE 1:**
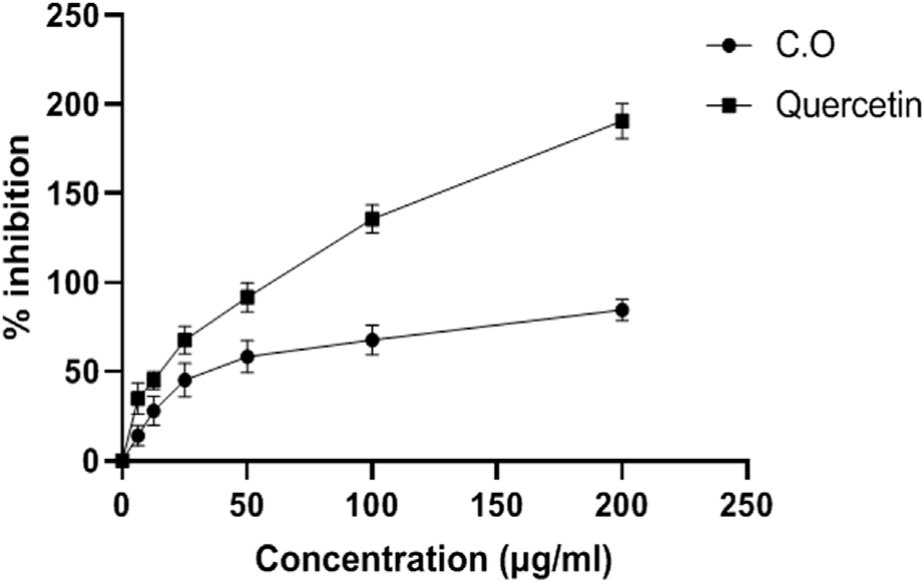
Antioxidant DPPH radical scavenging activity of aqueous methanolic extract of *C. odorata* (*in vitro*). Values are stated as mean ± SEM (*n* = 6).

Aqueous methanolic extract of *C. odorata* was found to be a promising reducing agent with an IC_50_ value of 78.38 ± 0.79 mg/ml. *C. odorata* extract showed 20.86 ± 0.87 U/mg protein of H_2_O_2_% inhibition. The DPPH % age activity of *C. odorata* extract is shown in Figure 1. *C. odorata* extract showed % age DPPH inhibition with maximum effect of 84.61% at 200 μg/ml concentration. The IC_50_ value was 29.78 μg/ml, similar to the effect of quercetin (IC_50_ value = 107.3 μg/ml).

### FTIR analysis

**FIGURE 2:**
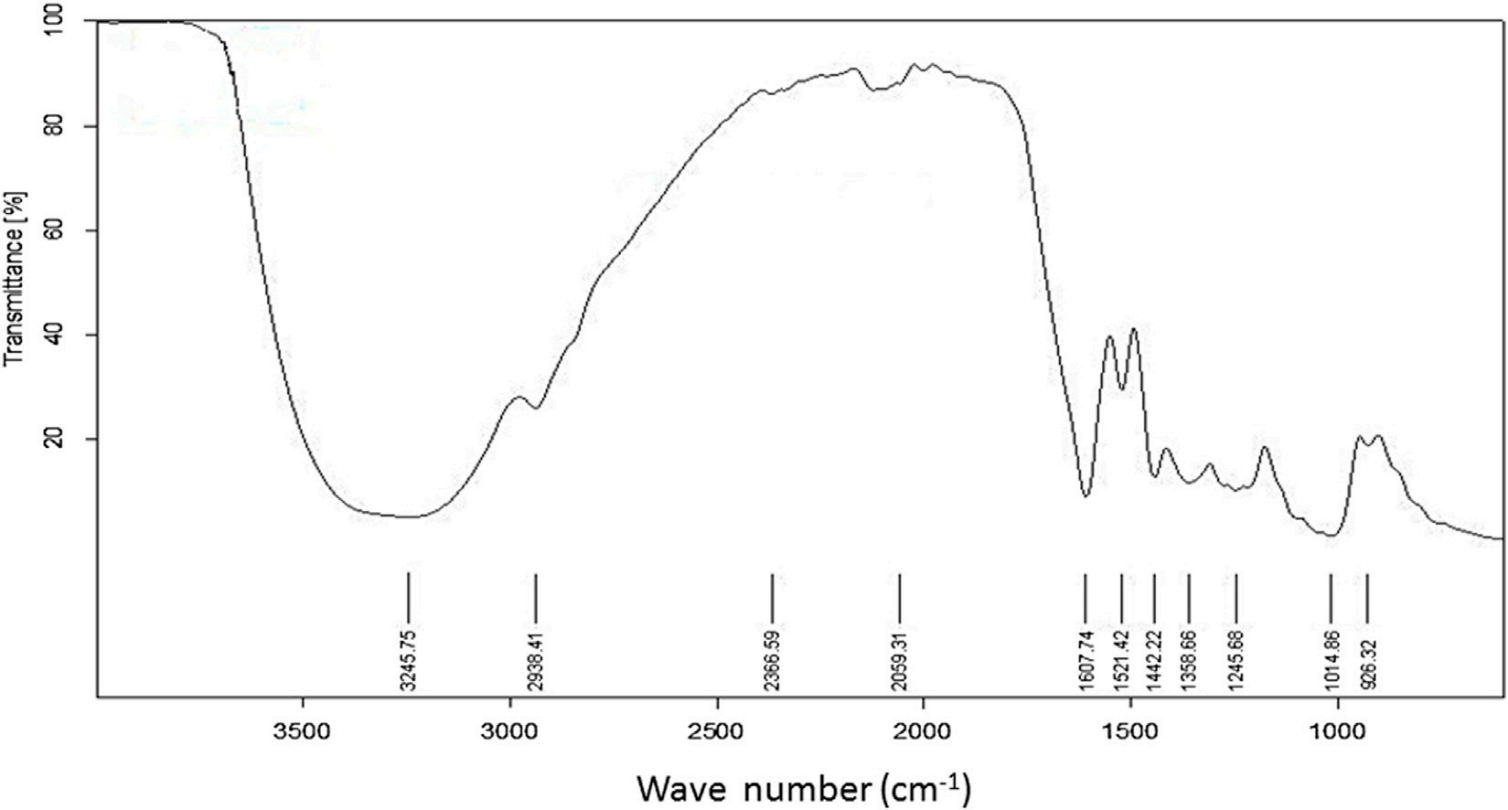
FTIR spectrum of aqueous methanolic extract of *C. odorata* studied at 400–4,000 cm^−1^ range.

Figure 2 depicts the spectrum of the FTIR analysis of *C. odorata* extract. The ATR-FTIR spectrum was interpreted in comparison with previous reported studies (Table 1). An infrared spectrum represents a fingerprint of a sample with absorption peaks which correspond to the frequencies of vibrations between the bonds of the atoms comprising the material. The absorption signals of 1–11 wave numbers in *C. odorata* FTIR-spectrum showed first peak at 3,245.75 cm^−1^, which lies in the reference range of 3,000–3,600. This range represents possible stretching of C–H, O–H and N–H bands of alcohol, phenol, amine, or amide. The second peak of 2,938.41 cm^−1^ found in the 2,800–2,900 reference range indicates a C–H stretch of alkanes. The peak at 2,366.59 cm^−1^ and 2059.31 cm^−1^ at a 2000–2,500 reference range represents C≡N and C≡C bonds of alkynes and isothiocyanate. Peak 5 at 1,607.74 cm^−1^ of the 1,600–1706 range revealed the presence of amino acids. The peak of 1,521.24 at the 1,500–1,600 range finds N–H bonds of either carboxylic acid salt, amide of amino acids, or nitro compounds. The remaining peak at 1,442.22, 1,358.66, 1,245.68, 1,014.86, and 926.32 wavenumber (cm^−1^) indicates the aromatic or phenyl group, amide I, phenol, alcohol or nitro, alkyl halide, acyl, or phenyl, ether or aliphatic phosphate, and the methylene (CH_2_) group of isoprenoids as functional groups, respectively, as detailed in Figure 2 and Table 1.

**TABLE 1:** FTIR frequency range with peaks and functional groups present in aqueous methanolic extract of *C. odorata*.

No.	Wavenumber cm^−1^ (observed)	Wavenumber cm^−1^ (references)	Biomolecular assignment	Possible functional group	References
1	3,245.75	3,000–3,600	Stretching of C–H, O–H, and N–H bands	Water, phenols, protein, carbohydrates, alcohols, peroxides	Caunii et al. (2012), Cao et al. (2017)
2	2,938.41	2,800–2,900	C–H stretch	Lipids, metoxy derivatives (cis double bonds)	Caunii et al. (2012)
		2,970–2,950/2,880–2,860			
3	2,366.59, 2059.31	2000–2,500	C≡N, C≡C	Alkynes, isothiocyanate	Nandiyanto et al. (2019), Nnorom and Onuegbu (2019)
4	1,607.74	1,600–1760, 1,600–1706	Aromatic ring stretch, vibrations N-H, C=O, C–O, C–N, CNN	Aldehydes, cetones, esters, amino acids, fatty acids, and ester-like glycerides	Caunii et al. (2012), Hands et al. (2016), Tatarua L.D (2017)
5	1,521.24	1,500–1,600	N–H bending vibrations, carboxylic acid salt, amide	Amino acids	Caunii et al. (2012), Tatarua L.D (2017)
6	1,442.22	1,380–1,465	Stretching vibrations CO and C-C, prim, sec or teri OH, phenol	Amide II, phenyl groups	Hands et al. (2016)
7	1,358.66	1,315–1,384	CH_3_/CH_2_ bending	Amide I	Hands et al. (2016)
8	1,245.68	1,150–1,270	Tertiary amine, CN stretch, C-O vibrations	Acid or ester	Caunii et al. (2012), Tatarua (2017)
9	1,014.86	997–1,130	Aliphatic phosphates (P–O–C stretch)	(Mono-, oligo-, and carbohydrates) deoxyribose/ribose, DNA, RNA (PO^2-^)	Caunii et al. (2012), Hands et al. (2016)
		1,008–1,230	C–C stretch, C–H bend		
10	926.32	<1,000	Methylene– (CH2) n, trans-C–H, cis–C–H	Isoprenoids	Caunii et al. (2012)

### HPLC analysis

**FIGURE 3:**
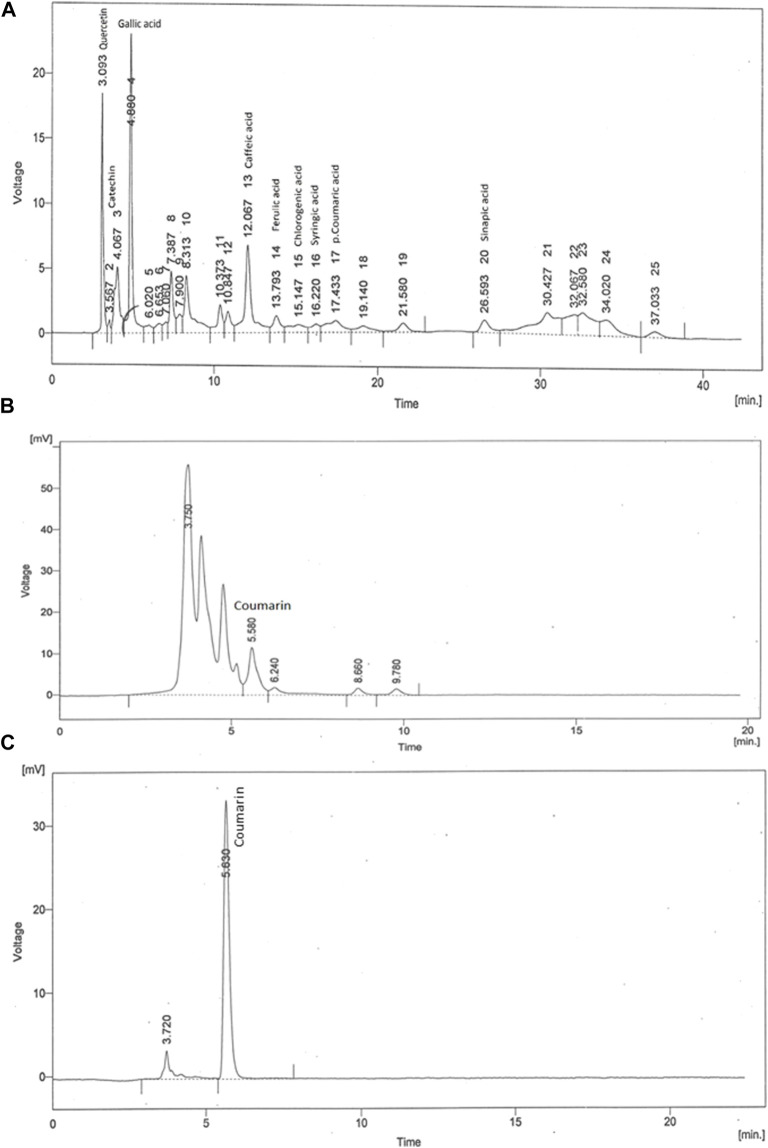
HPLC fingerprint of **(A)** aqueous methanolic extract of *C. odorata* in gradient mode, **(B)** aqueous methanolic extract of *C. odorata* in isocratic mode, and **(C)** standard coumarin.

The achieved chromatograms of *C. odorata* extract (Figures 3A, B) showed identified compounds as plant constituents. The HPLC profiles of the test materials were related to the standards. The quantitative results were presented in ppm, where quercetin (7.53), catechin (0.34), gallic acid (9.42), caffeic acid (8.75), ferulic acid (176.6), chologenic acid (3.23), syringic acid (0.58), p-coumaric acid (0.84), sinapic acid (0.39) (Figure 3A), and coumarins (416.24) (Figure 3B) were found as active ingredients of *C. odorata*. The observed retention times (rt) of these standards were 3.09, 3.56, 4.88, 12.06, 13.79, 15.14, 16.22, 17.43, and 26.59 min, respectively. Standard coumarin appeared with 5.63 min (rt) (Figure 3C), while it appeared in plant extract at a retention time of 5.58 min.

### Effect of *C. odorata* and coumarin on body weight, feed intake, and weight of different body organs

**FIGURE 4:**
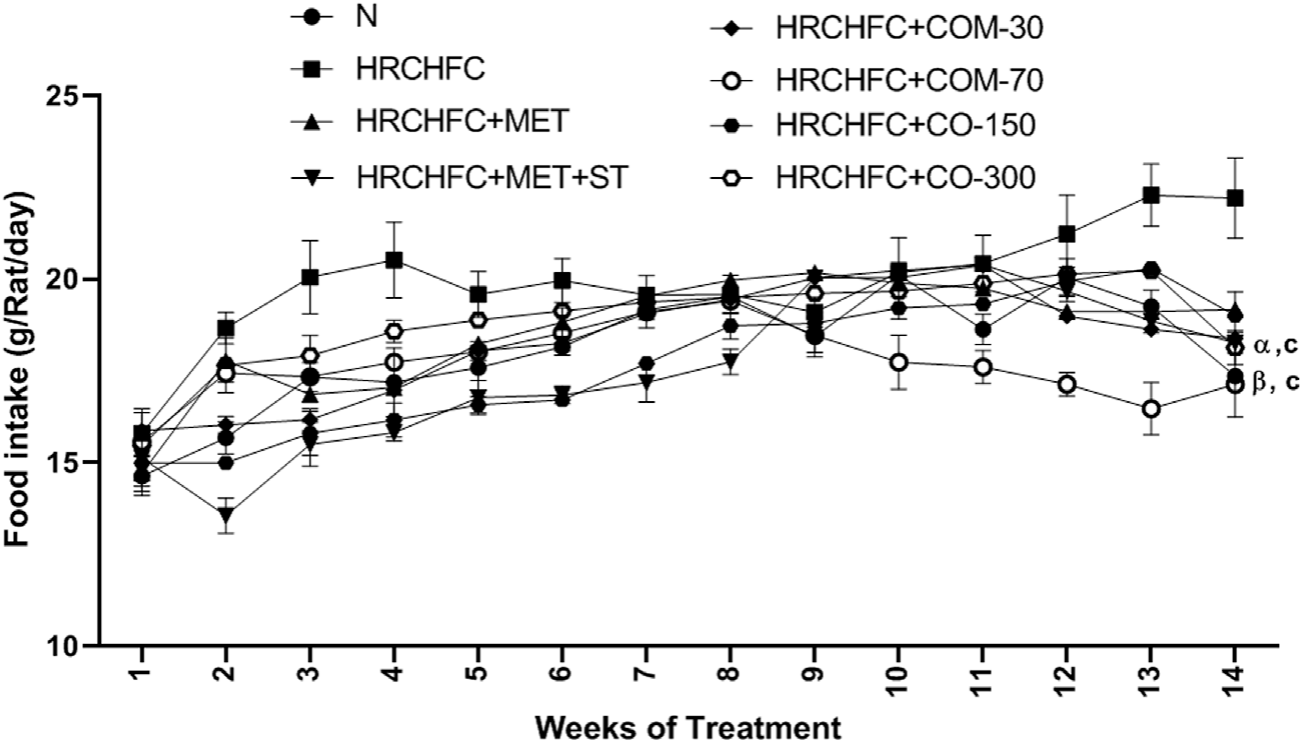
Effect of *C. odorata* and coumarin administration on feed intake in HRCHFC- diet induced CMS rats. N: normal control; HRCHFC: high refined carbohydrate-high fat-cholesterol loaded diet induced CMS control; HRCHFC + MET: (metformin 300 mg/kg); HRCHFC + MET + ST: (metformin and rosuvastatin 200 mg/kg + 1.5 mg/kg); HRCHFC + COM-30: (coumarin 30 mg/kg); HRCHFC + COM-70: (coumarin 70 mg/kg); HRCHFC + CO-150: (*C. odorata* 150 mg/kg); HRCHFC + CO-300: (*C. odorata* 300 mg/kg). Values are expressed as mean ± SEM (*n* = 6), where ***p < 0.001, **p < 0.01, *p < 0.05 vs. normal control and ^c^ *p* < 0.001, ^b^ *p* < 0.01 and ^a^ *p* < 0.05 vs. CMS control group using two-way ANOVA followed by Bonferroni *post hoc* test. ns = non-significant, ^γ^ *p* < 0.001, ^β^ *p* < 0.01, ^α^ *p* < 0.05 shows effect of low vs. high dose of *C. odorata* and coumarin (Student’s t-test).

Initially, rats in all groups did not show a significant (*p* > 0.05) difference in their body weight. However, HRCHFC-fed rats showed a significant (*p* < 0.001) rise at Weeks 10–14. Chronic administration of *C. odorata*, coumarin or positive controls to HRCHFC-fed rats markedly (*p* < 0.001) reduced weight gain at Weeks 12–14 of the study (Table 2). Similarly, *C. odorata* treatment caused a marked (*p* < 0.001) reduction in feed intake pattern at Weeks 11–14 compared to HRCHFC-fed rat data. Coumarin or positive controls groups showed a consistent pattern of feed intake at the start of experiment and showed significant (*p* < 0.001) reduction in daily diet intake at Weeks 12–14 of the study period compared to HRCHFC group data (Figure 4). Table 3 shows that HRCHFC-fed rats displayed a marked (*p* < 0.001) increase in liver and heart weight with no effect on kidney weight at end of the study. Significant (*p* < 0.001) reductions in liver and heart weight were seen in all treatment groups.

**TABLE 2:** Effect of *C. odorata* and coumarin administration on body weight in HRCHFC- diet induced CMS rats.

Parameter	Duration (W)	Groups							
		N	HRCHFC	HRCHFC + MET	HRCHFC + MET + ST	HRCHFC + COM-30	HRCHFC + COM-70	HRCHFC + CO-150	HRCHFC + CO-300
Body weight (g)	0	135.6 ± 9.0	139.6 ± 5.9	157.6 ± 6.6	146.0 ± 13.9	155.4 ± 10.7	137.2 ± 4.3	168.2 ± 3.8	127.4 ± 8.1
	2	143.2 ± 13.5	148.0 ± 7.5	167.2 ± 2.8	158.2 ± 10.0	167.8 ± 9.7	148.6 ± 5.8	179.0 ± 3.1	143.2 ± 6.9
	4	155.4 ± 12.7	164.4 ± 8.5	177.8 ± 4.0	176.2 ± 10.5	176.0 ± 12.1	154.8 ± 11.7	195.4 ± 5.7^*^	155.4 ± 6.7
	6	164.6 ± 14.3	184.0 ± 6.6	187.2 ± 12.3	182.6 ± 7.3	184.8 ± 13.0	166.8 ± 8.3	204.0 ± 7.7	162.4 ± 6.4
	8	183.8 ± 10.4	221.20 ± 8.5	194.0 ± 5.3	198.6 ± 7.0	194.8 ± 12.5	181.2 ± 6.5^a*^	209.4 ± 8.2	185.2 ± 7.2
	10	196.0 ± 10.1^c^	257.4 ± 0.4^***^	209.2 ± 13.9^**b^	210.0 ± 7.8^**b^	209.0 ± 9.9^**b^	209.4 ± 22.4^**b/ns^	218.8 ± 10.2^*a^	201.8 ± 7.8^***c/ns^
	12	201.4 ± 8.7^c^	281.8 ± 6.8^***^	212.6 ± 11.7^***c^	211.2 ± 9.1^***c^	221.4 ± 13.1^***c^	212.8 ± 25.6^***c/ns^	220.6 ± 5.2^***c^	202.8 ± 6.0^***c/α^
	14	207.6 ± 7.2^c^	308.0 ± 5.5^***^	223.2 ± 20.7^***c^	218.8 ± 7.3^***c^	224.0 ± 0.3^***c^	214.4 ± 0.5^***c/γ^	222.4 ± 4.7^***c^	192.6 ± 5.8^***c/β^

Effect of *C. odorata* and coumarin administration on body weight in HRCHFC-diet induced CMS rats. N: normal control; HRCHFC: high refined carbohydrate-high fat-cholesterol loaded diet induced CMS control; HRCHFC + MET: (metformin 300 mg/kg); HRCHFC + MET + ST: (metformin and rosuvastatin 200 mg/kg +1.5 mg/kg); HRCHFC + COM-30: (coumarin 30 mg/kg); HRCHFC + COM-70: (coumarin 70 mg/kg); HRCHFC + CO-150: (*C. odorata* 150 mg/kg); HRCHFC + CO-300: (*C. odorata* 300 mg/kg). Values are stated as mean ± SEM (*n* = 6), where ***p < 0.001, **p < 0.01, *p < 0.05 vs. normal control and ^c^ *p* < 0.001, ^b^ *p* < 0.01, and ^a^ *p* < 0.05 vs. CMS control group using two-way ANOVA followed by Bonferroni *post hoc* test. ns = non-significant, ^γ^ *p* < 0.001, ^β^ *p* < 0.01, ^α^ *p* < 0.05 shows effect of low vs. high dose of *C. odorata* and coumarin in treatment period (Student’s t-test).

**TABLE 3:** Effect of *C. odorata* and coumarin administration on weight of various organs, feed intake, and fasting blood glucose level in HRCHFC-diet induced CMS rats.

Parameters	N	HRCHFC	HRCHFC + MET	HRCHFC + MET + ST	HRCHFC + COM-30	HRCHFC + COM-70	HRCHFC + CO-150	HRCHFC + CO-300
Liver. Wt. (g)	7.8 ± 0.1^c^	13.9 ± 0.0^***^	11.5 ± 0.2***^c^	8.9 ± 0.1 ***^c^	10.7 ± 0.2***^c^	8.8 ± 0.2***^c/γ^	11.2 ± 0.4**^c^	9.8 ± 0.5**^c/α^
Kidney. wt. (g)	0.98 ± 0.0	1.0 ± 0.0	1.1 ± 0.0	0.99 ± 0.0	0.94 ± 0.0	0.98 ± 0.0^ns^	1.0 ± 0.0	0.99 ± 0.0^ns^
Heart. wt. (g)	0.81 ± 0.0^c^	1.3 ± 0.03^***^	0.86 ± 0.0^c^	0.80 ± 0.0^c^	0.82 ± 0.0^c^	0.79 ± 0.0^c/ns^	1.0 ± 0.0^c^	0.95 ± 0.0^c/ns^
Feed intake (g/rat/d)	17.1 ± 0.9^c^	21.2 ± 0.5^*^	18.5 ± 0.0^a^	17.5 ± 0.0^b^	18.3 ± 0.0^b^	17.6 ± 0.2^b/α^	17.9 ± 0.2^b^	17.1 ± 0.2^c/β^
FBG (mg/dl)	82.0 ± 6.3	134.6 ± 3.5^**^	87.6 ± 6.9^c^	73.0 ± 4.7^c^	95.3 ± 3.7^c^	86.3 ± 0.8^c/α^	96.6 ± 4.0^c^	79.6 ± 6.0^c/α^

Effect of *C. odorata* and coumarin administration on weight of various organs, feed intake, and fasting blood glucose (FBG) level in HRCHFC-diet induced CMS rats. Values are stated as mean ± SEM (*n* = 6), where ^ns^ = non-significant, ***p < 0.001, **p < 0.01, *p < 0.05 vs. normal control and ^c^ *p* < 0.001, ^b^ *p* < 0.01 and ^a^ *p* < 0.05 vs. CMS control group using one-way ANOVA followed by Dunnett’s test. ns = non-significant, ^γ^ *p* < 0.001, ^β^ *p* < 0.01, ^α^ *p* < 0.05 shows effect of low vs. high dose of *C. odorata* and coumarin (Student’s t-test).

### Hypotensive effect of *C. odorata* and coumarin

The hypotensive effect of chronically-administered *C. odorata* and coumarin on systolic blood pressure, mean arterial pressure, diastolic blood pressure, and heart rate in HRCHFC-fed rats is evident in Tables 4 and 5. The results clearly showed that HRCHFC diet administration revealed a noticeable (*p* < 0.001) rise in SBP, MAP, and DBP, along with HR, at Weeks 12–14 of the study period compared to the control rats. Subsequently, after 6 weeks of the treatment schedule, all treated rats manifested a significant (*p* < 0.001) decline in blood pressure (SBP, DBP, MBP) and HR at Weeks 10–14 of the study period compared to HRCHFC-fed rats. The hypotensive effect of *C. odorata* and coumarin were observed dose-dependent in SBP, MBP (10–14 weeks), and HR (12–14 weeks).

**TABLE 4:** Effect of *C. odorata* and coumarin administration on systolic blood pressure (SBP), mean blood pressure (MBP), and diastolic blood pressure (DBP) in HRCHFC-diet induced CMS rats.

Parameters	Duration (W)	Groups							
		N	HRCHFC	HRCHFC + MET	HRCHFC + MET + ST	HRCHFC + COM-30	HRCHFC + COM-70	HRCHFC + CO-150	HRCHFC + CO-300
SBP (mmHg)	0	123.0 ± 1.5	122.3 ± 1.6	123.8 ± 2.7	119.0 ± 0.5	120.6 ± 1.2	115.3 ± 0.8^a^*	121.96 ± 2.7	122.6 ± 1.9
	4	125.5 ± 0.8^c^	149.9 ± 0.9^***^	145.2 ± 0.6^***^	144.4 ± 1.2^***^	149.7 ± 1.3^***^	144.1 ± 0.9^***^	144.4 ± 2.9^***^	145.8 ± 2.1^***^
	8	124.3 ± 0.7^c^	178.9 ± 3.4^***^	176.6 ± 2.3^***^	176.3 ± 0.6^***^	177.7 ± 1.0^***^	174.7 ± 1.4^***^	176.0 ± 1.3^***^	175.7 ± 3.3^***^
	10	120.3 ± 0.9^c^	180.0 ± 2.3^***^	139.4 ± 2.3^***c^	134.0 ± 1.0^***c^	155.3 ± 1.2^***c^	158.2 ± 2.1^***c/ns^	157.6 ± 3.7^***c^	148.2 ± 2.0^***c/α^
	12	125.3 ± 0.9^c^	181.9 ± 1.4^***^	129.3 ± 0.6^c^	126.4 ± 1.0^c^	136.8 ± 0.9^***c^	119.6 ± 0.8^c/γ^	126.2 ± 1.0^c^	118.2 ± 2.4*^c/α^
	14	126.7 ± 0.7^c^	182.4 ± 1.4^***^	119.6 ± 0.8*^c^	117.3 ± 0.6^**c^	108.7 ± 0.9^***c^	103.2 ± 1.0^***c/β^	112.8 ± 2.0^***c^	99.1 ± 1.3^***c/γ^
MBP (mmHg)	0	86.1 ± 1.6^c^	97.4 ± 1.1^***^	95.2 ± 0.5^**^	87.4 ± 1.1^c^	92.3 ± 1.6	90.2 ± 1.1^a^	90.1 ± 1.2^a^	94.4 ± 0.6^**^
	4	99.8 ± 0.5^c^	115.5 ± 1.9^***^	119.1 ± 2.1^***^	119.3 ± 0.5^***^	118.3 ± 2.5^***^	114.6 ± 3.9^***^	117.5 ± 0.6^***^	113.4 ± 2.6^***^
	8	97.4 ± 1.1^c^	124.2 ± 0.5^***^	123.7 ± 1.4^***^	125.5 ± 1.9^***^	124.6 ± 2.6^***^	125.6 ± 0.9^***^	125.4 ± 2.1^***^	124.9 ± 1.3^***^
	10	95.2 ± 0.0^c^	125.8 ± 0.8^***^	105.2 ± 0.5^***c^	106.3 ± 0.5^***c^	109.3 ± 0.5^***c^	115.3 ± 1.7^***c/β^	117.4 ± 4.5^***b^	118.5 ± 1.2^***a/ns^
	12	93.4 ± 0.1^c^	126.3 ± 0.5^***^	95.2 ± 0.5^c^	95.6 ± 0.7^c^	103.1 ± 1.1^***c^	97.0 ± 1.5^c/β^	107.6 ± 3.2^***c^	100.1 ± 1.0^*c/α^
	14	92.3 ± 1.6^c^	127.1 ± 0.6^***^	92.0 ± 1.4^c^	100.1 ± 0.5^**c^	94.8 ± 1.1^c^	92.3 ± 0.0^c/α^	95.2 ± 0.6^c^	87.2 ± 1.1^c/γ^
DBP (mmHg)	0	67.6 ± 1.0^c^	85.0 ± 0.4^***^	80.8 ± 0.7^***^	71.6 ± 0.5^c^	78.1 ± 0.5^***b^	77.6 ± 0.7^***c^	74.2 ± 0.6^**c^	80.3 ± 0.3^***a^
	4	87.0 ± 0.9^c^	98.3 ± 0.8^***^	106.0 ± 1.6^***c^	106.7 ± 1.6^***c^	102.5 ± 1.2^***^	99.9 ± 0.3^***^	104.0 ± 0.8^***b^	97.2 ± 0.9^***^
	8	84.3 ± 2.0^c^	96.5 ± 0.0^***^	97.1 ± 0.6^***^	100.7 ± 0.4^***^	98.7 ± 0.4^***^	101.6 ± 0.7^***a^	100.4 ± 0.5^***^	99.5 ± 0.5^***^
	10	82.6 ± 0.0^c^	98.3 ± 0.0^***^	88.1 ± 2.5^*c^	92.5 ± 2.6^***a^	86.2 ± 0.8^c^	93.6 ± 0.6^***/γ^	97.3 ± 1.1^***^	103.6 ± 0.8^***/γ^
	12	77.4 ± 0.7^c^	99.8 ± 0.4^***^	78.2 ± 0.4^c^	80.2 ± 2.2^c^	87.2 ± 0.6^***c^	85.7 ± 3.2^***c/ns^	98.3 ± 0.9^***b^	91.0 ± 0.6^***c/γ^
	14	75.1 ± 1.3^c^	108.9 ± 1.7^***^	78.1 ± 0.0^c^	91.2 ± 3.1^***c^	87.8 ± 0.2^***c^	86.3 ± 2.8^***c/ns^	86.4 ± 0.4^***c^	81.3 ± 0.8^**c/β^

Effect of *C. odorata* and coumarin administration on systolic blood pressure (SBS), mean blood pressure (MBP) and diastolic blood pressure in HRCHFC-diet induced CMS rats. Values are stated as mean ± SEM (*n* = 6), where ***p < 0.001, **p < 0.01, *p < 0.05 vs. normal control and ^c^ *p* < 0.001, ^b^ *p* < 0.01 and ^a^ *p* < 0.05 vs. CMS control group using two-way ANOVA followed by Bonferroni *post hoc* test. ns = non-significant, ^γ^ *p* < 0.001, ^β^ *p* < 0.01, ^α^ *p* < 0.05 shows effect of low vs. high dose of *C. odorata* and coumarin in treatment period (Student’s t-test).

**TABLE 5:** Effect of *C. odorata* and coumarin administration on heart rate in HRCHFC-diet induced CMS rats.

Groups									
Parameter	Duration (W)	N	HRCHFC	HRCHFC + MET	HRCHFC + MET + ST	HRCHFC + COM-30	HRCHFC + COM-70	HRCHFC + CO-150	HRCHFC + CO-300
Heart rate (BPM)	0	299.4 ± 11.6	309.1 ± 6.5	289.5 ± 7.5	304.6 ± 3.9	304.1 ± 7.3	308.4 ± 5.4	291.8 ± 11.4	295.4 ± 10.0
	4	314.2 ± 7.9^c^	388.9 ± 5.8^***^	375.9 ± 5.7^***^	385.1 ± 7.6^***^	376.5 ± 5.1^***^	378.1 ± 6.1^***^	383.5 ± 7.0^***^	372.1 ± 11.8^***^
	8	328.9 ± 3.4^c^	425.06 ± 2.2^***^	419.7 ± 3.4^***^	430.7 ± 3.0^***^	439.4 ± 0.5^***^	432.3 ± 2.0^***^	429.5 ± 8.2^***^	436.6 ± 5.2^***^
	10	319.7 ± 10.2^c^	446.4 ± 2.0^***^	367.7 ± 5.6^***c^	364.1 ± 9.9^**c^	373.3 ± 9.9^***c^	366.8 ± 7.5^***c/ns^	383.6 ± 11.2^***c^	379.7 ± 2.1^***c/ns^
	12	320.9 ± 3.1^c^	452.6 ± 0.9^***^	332.0 ± 8.3^c^	326.6 ± 2.1^c^	341.8 ± 0.3^c^	331.5 ± 3.8^c/α^	354.3 ± 7.7^*c^	302.9 ± 14.6^c/β^
	14	309.6 ± 2.3^c^	485.7 ± 5.8^***^	312.8 ± 6.1^c^	303.5 ± 9.0^c^	318.4 ± 1.6^c^	296.2 ± 3.7^c/γ^	287.5 ± 0.4^c^	279.2 ± 1.1^c/γ^

Effect of *C. odorata* and coumarin administration on heart rate (HR) in HRCHFC-diet induced CMS rats. Values are expressed as mean ± SEM (*n* = 6), where ***p < 0.001, **p < 0.01, *p < 0.05 vs. normal control and ^*c*^ *p <* 0.001, ^*b*^ *p* < 0.01, and ^*a*^ *p* < 0.05 vs. CMS control group using two-way ANOVA followed by Bonferroni *post hoc* test. ns = non-significant, ^γ^ *p* < 0.001, ^β^ *p* < 0.01, ^α^ *p* < 0.05 shows effect of low vs. high dose of *C. odorata* and coumarin in treatment period (Student’s t-test).

### Effect of *C. odorata* and coumarin on fasting blood glucose (FBG), oral glucose tolerance (OGT), and insulin sensitivity tests (ITT)

**FIGURE 5:**
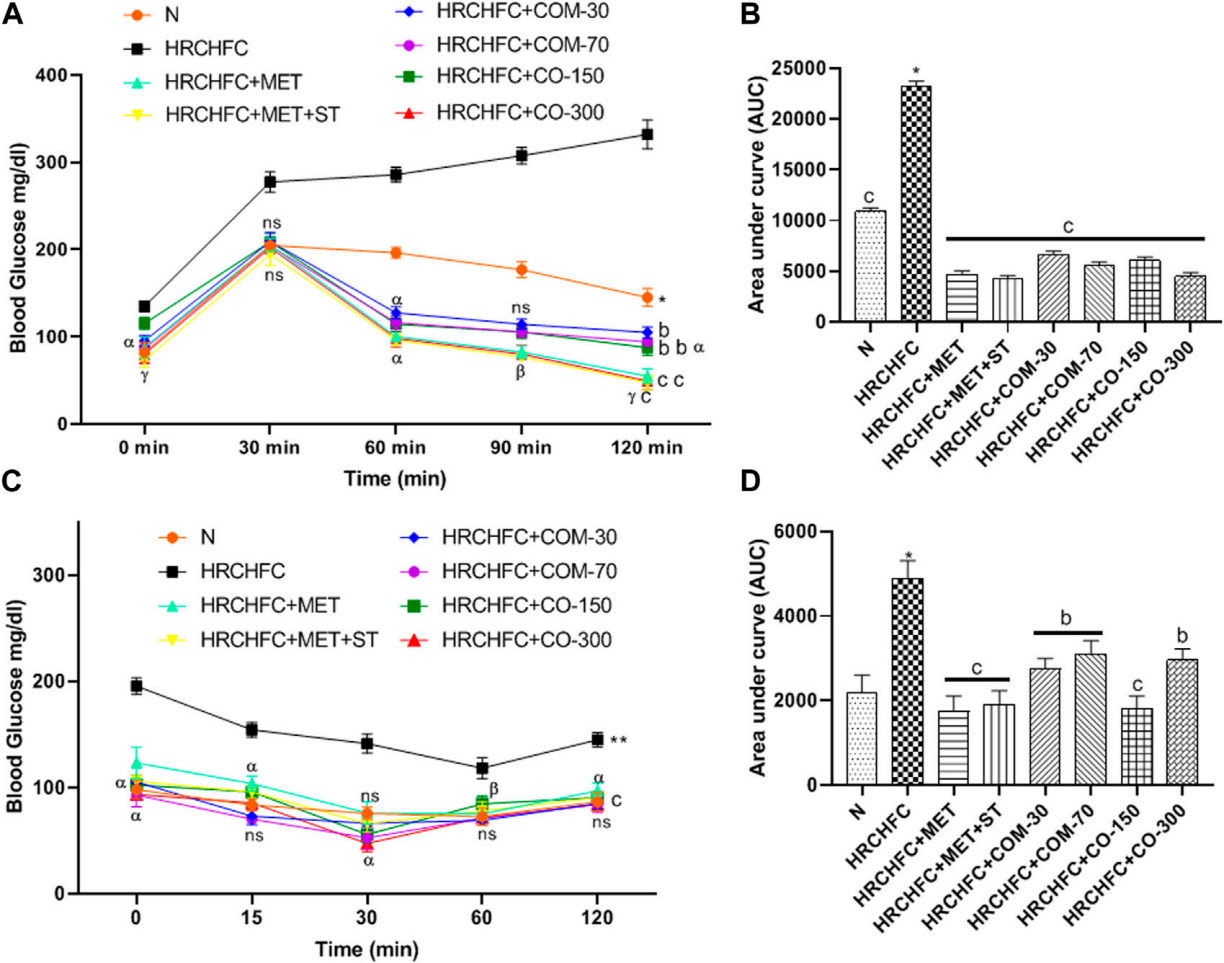
Effect of *C. odorata* and coumarin administration on **(A)** oral glucose tolerance test during 14th week of study, **(B)** AUC of blood glucose level of animals measured at end of treatment, **(C)** insulin tolerance test, and **(D)** AUC of blood glucose level of animals measured after insulin injection in HRCHFC diet induced CMS rats. N: normal control; HRCHFC: high refined carbohydrate-high fat-cholesterol loaded diet induced CMS control; HRCHFC + MET: (metformin 300 mg/kg); HRCHFC + MET + ST: (metformin and rosuvastatin 200 mg/kg +1.5 mg/kg); HRCHFC + COM-30: (coumarin 30 mg/kg); HRCHFC + COM-70: (coumarin 70 mg/kg); HRCHFC + CO-150: (*C. odorata* 150 mg/kg); HRCHFC + CO-300: (*C. odorata* 300 mg/kg). Values are expressed as mean ± SEM (*n* = 6), where ***p < 0.001, **p < 0.01, *p < 0.05 shows normal control vs. CMS control group (Student’s t-test); ^c^ *p* < 0.001, ^b^ *p* < 0.01 and ^a^ *p* < 0.05 show a comparison of treatment vs. CMS control group using one-way ANOVA followed by Dennett’s test. ns = non-significant, ^γ^ *p* < 0.001, ^β^ *p* < 0.01, ^α^ *p* < 0.05 shows effect of low vs. high dose of *C. odorata* and coumarin (Student’s t-test).

Administration of *C. odorata* and coumarin significantly (*p* < 0.001) showed a dose-dependent reduction in elevated fasting blood glucose in HRCHFC-fed rats (Table 3). These effects were comparable to standards. HRCHFC-fed rats exhibited notably (*p* < 0.05) impaired glucose tolerance against normal control rats. A remarkably (*p* < 0.001) faster disposal of glucose levels was noticed from circulation at 60, 90, and 120 min in *C. odorata* and coumarin-treated rats compared to HRCHFC-fed rats (Figure 5A). The AUC study revealed a significant difference in HRCHFC-fed rats compared to normal control rats (Figure 5B **)**. The ITT results showed that, after 6 weeks of treatment with *C. odorata* and coumarin, significant (*p* < 0.001) differences in blood glucose levels were analyzed following insulin administration. ITT showed notably (*p* < 0.01) lower responsiveness to insulin in HRCHFC-fed rats compared to normal rats. *C. odorata* and coumarin-treated rats showed marked (*p* < 0.001) insulin sensitivity compared to HRCHFC-fed rats. These results were verified by AUC analysis between treated groups during the experiment period (Figures 5C, D).

### Effect of *C. odorata* and coumarin on serum biochemical markers

HRCHFC-fed rats showed a significant (*p* < 0.001) increase in serum triglycerides and total cholesterol (lipid biomarkers), aspartate transaminase, alanine transaminase (liver biomarker enzyme), urea, and creatinine (renal biomarkers) levels, while showing a notable reduction in serum high density lipoprotein cholesterol levels compared to the normal control group. However, concurrent treatment with *C. odorata*, coumarin, and positive controls produced a notable (*p* < 0.001) decline in serum TC, TG, ALT, AST, urea, and creatinine levels, while elevated HDL-C levels were observed compared to only HRCHFC-fed rats (Table 6).

**TABLE 6:** Effect of *C. odorata* and coumarin administration on biochemical markers in HRCHFC-diet induced CMS rats.

Groups	Triglycerides (mg/dl)	HDL (mg/dl)	Cholesterol (mg/dl)	ALT (U/L)	AST (U/L)	Creatinine (mg/dl)	Urea (mg/dl)
N	81.4 ± 1.2^c^	61.4 ± 1.7^c^	70.0 ± 1.2^c^	30.6 ± 1.3^c^	68.6 ± 5.7^b^	0.6 ± 0.0^c^	32.6 ± 1.8^c^
HRCHFC	205.0 ± 3.4^***^	32.8 ± 3.4^***^	262.6 ± 3.0^***^	96.1 ± 3.4^***^	106.2 ± 9.7^**^	1.8 ± 0.3^***^	74.7 ± 6.7^***^
HRCHFC + MET	97.7 ± 6.7^*c^	68.2 ± 1.7^c^	78.3 ± 1.7^c^	45.7 ± 6.2^c^	88.7 ± 0.7	0.7 ± 0.0^c^	30.3 ± 0.7^c^
HRCHFC + MET + ST	90.6 ± 0.5^c^	71.3 ± 4.0^c^	69.2 ± 2.0^c^	35.7 ± 7.1^c^	71.7 ± 2.9^b^	0.5 ± 0.0^c^	18.4 ± 0.5^*c^
HRCHFC + COM-30	85.7 ± 1.9^c^	55.5 ± 3.2^b^	98.1 ± 1.7^***c^	36.0 ± 4.1^c^	75.7 ± 6.2^b^	0.8 ± 0.0^c^	34.2 ± 0.3^c^
HRCHFC + COM-70	67.8 ± 4.1^c/β^	68.7 ± 1.9^c/β^	76.3 ± 3.5^c/γ^	33.0 ± 1.2^c/ns^	60.5 ± 2.6^c/α^	0.6 ± 0.0^c/γ^	25.4 ± 0.7^c/γ^
HRCHFC + CO-150	95.0 ± 1.4^c^	45.6 ± 3.5^*^	71.7 ± 1.5^c^	32.7 ± 1.8^c^	54.5 ± 3.6^c^	0.9 ± 0.0^c^	34.8 ± 2.4^c^
HRCHFC + CO-300	85.1 ± 2.1^c/β^	63.4 ± 2.4^c/β^	59.7 ± 3.4^c/β^	23.8 ± 0.8^c/β^	37.7 ± 5.6^**c/α^	0.7 ± 0.0^c/β^	33.4 ± 0.8^c/ns^

Effect of *C. odorata* and coumarin administration on biochemical markers in HRCHFC-diet induced CMS rats. Values are expressed as mean ± SEM (*n* = 6), where ***p < 0.001, **p < 0.01, *p < 0.05 vs. normal control and ^c^ *p* < 0.001, ^b^ *p* < 0.01, and ^a^ *p* < 0.05 vs. CMS control group using one-way ANOVA followed by Dunnett’s test. ns = non-significant, ^γ^ *p* < 0.001, ^β^ *p* < 0.01, ^α^ *p* < 0.05 shows effect of low vs. high dose of *C. odorata* and coumarin (Student’s t-test).

### Effect of *C. odorata* and coumarin on serum biomarkers of leptin, adiponectin, chemerin, and HMG-CoA reductase levels

**FIGURE 6:**
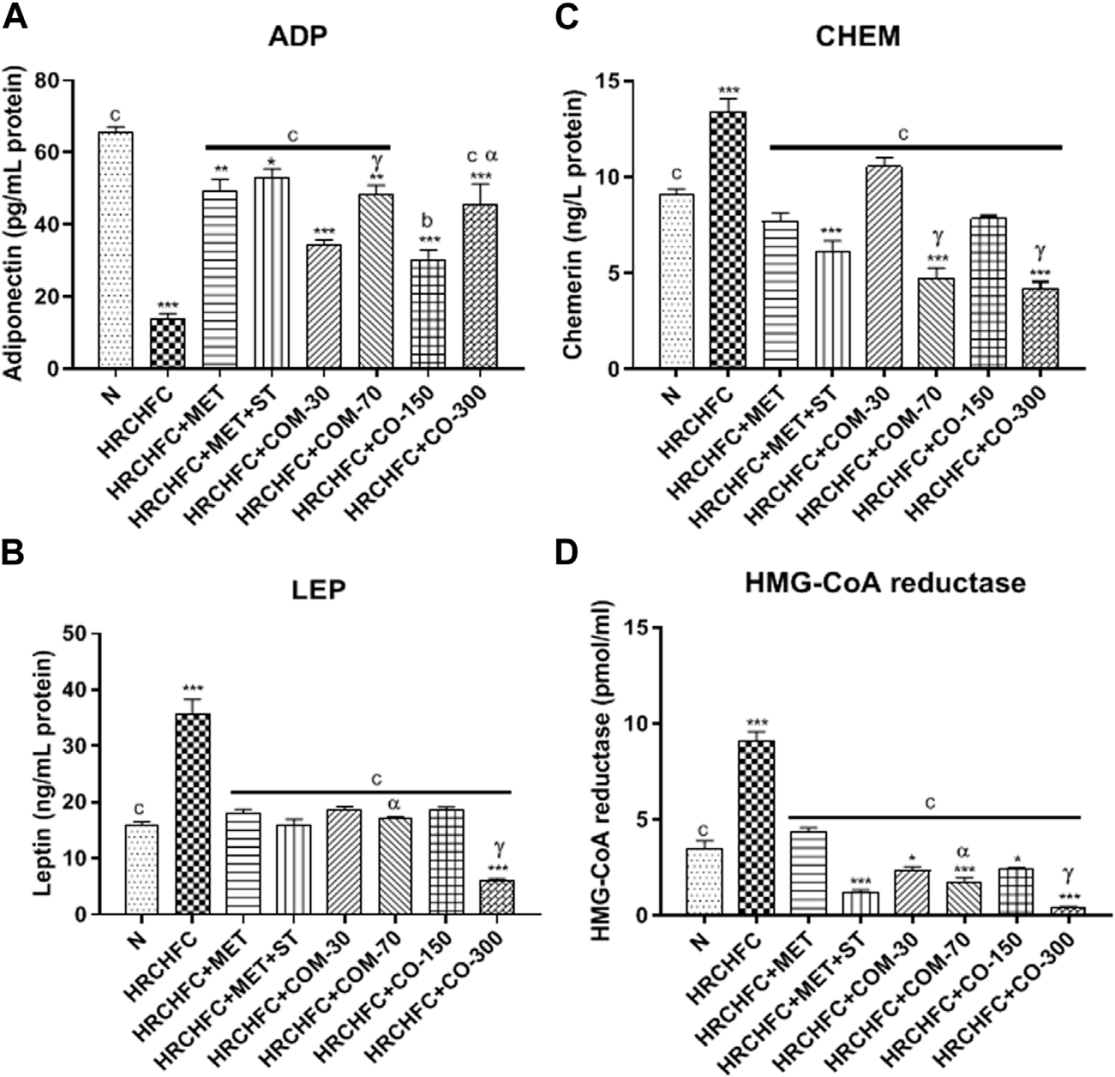
Effect of *C. odorata* and coumarin administration on serum **(A)** ADP, **(B)** LEP, **(C)** CHEM, **(D)** HMG-CoA reductase in HRCHFC-diet induced CMS rats. N: normal control; HRCHFC: high refined carbohydrate-high fat-cholesterol loaded diet induced CMS control; HRCHFC + MET: (metformin 300 mg/kg); HRCHFC + MET + ST: (metformin and rosuvastatin 200 mg/kg +1.5 mg/kg); HRCHFC + COM-30: (coumarin 30 mg/kg); HRCHFC + COM-70: (coumarin 70 mg/kg); HRCHFC + CO-150: (*C. odorata* 150 mg/kg); HRCHFC + CO-300: (*C. odorata* 300 mg/kg). Values are expressed as mean ± SEM (*n* = 6), where ***p < 0.001, **p < 0.01, *p < 0.05 vs. normal control, and ^c^ *p* < 0.001, ^b^ *p* < 0.01 and ^a^ *p* < 0.05 vs. CMS control group using one-way ANOVA followed by Dunnett’s test. ns = non-significant, ^γ^ *p* < 0.001, ^β^ *p* < 0.01, ^α^ *p* < 0.05 shows effect of low vs. high dose of *C. odorata* and coumarin (Student’s t-test).

The animals on the HRCHFC diet showed a marked (*p* < 0.001) elevation in serum leptin and chemerin levels and HMG-CoAreductase activity with serum leptin, chemerin and HMG-CoA reductase levels, while adiponectin levels were found to be decreased in diseased animals compared to the normal control group. After the sixth week of treatment with *C. odorata*, coumarin, and positive controls, there was a marked (*p* < 0.001) suppression in leptin and chemerin levels and HMG-CoAreductase activity with leptin, chemerin and HMG-CoA reductase levels, while a significant (*p* < 0.001) rise of adiponectin levels was observed in treated rats compared with data from only HRCHFC-fed rats (Figure 6).

### Effect of *C. odorata* and coumarin on inflammatory biomarkers (TNF-α and IL-6)

**FIGURE 7:**
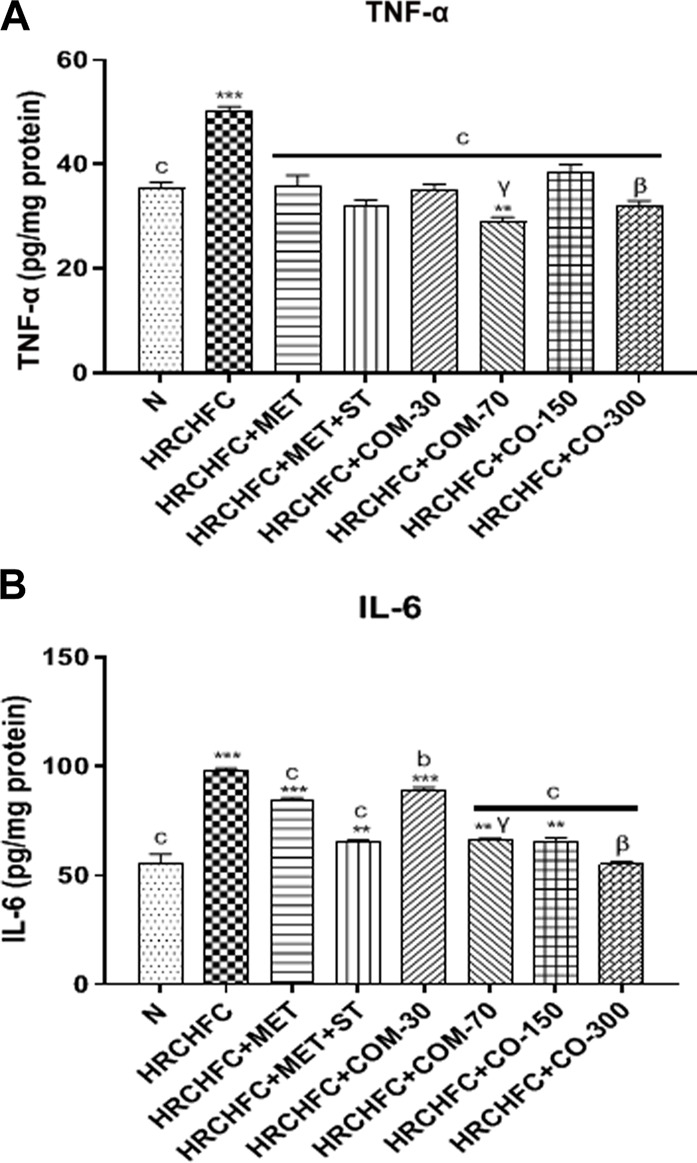
Effect of *C. odorata* and coumarin administration on serum **(A)** TNF-α, **(B)** IL-6 in HRCHFC diet induced CMS rats. Values are expressed as mean ± SEM (*n* = 6), where ***p < 0.001, **p < 0.01, *p < 0.05 vs. normal control and ^c^ *p* < 0.001, ^b^ *p* < 0.01 and ^a^ *p* < 0.05 vs. CMS control group using one-way ANOVA followed by Dunnett’s test. ns = non-significant, ^γ^ *p* < 0.001, ^β^ *p* < 0.01, ^α^ *p* < 0.05 shows effect of low vs. high dose of *C. odorata* and coumarin (Student’s t-test).

HRCHFC-fed rats displayed a significant (*p* < 0.001) elevation in TNF-α and IL-6 levels versus the normal control group. However, oral treatment of *C. odorata* and coumarin to diseased animals produced a significant (*p* < 0.001) fall in serum TNF-α and IL-6 levels to normal values, comparable to positive controls (Figure 7 **)**.

### Effect of *C. odorata* and coumarin on oxidative stress markers

**FIGURE 8:**
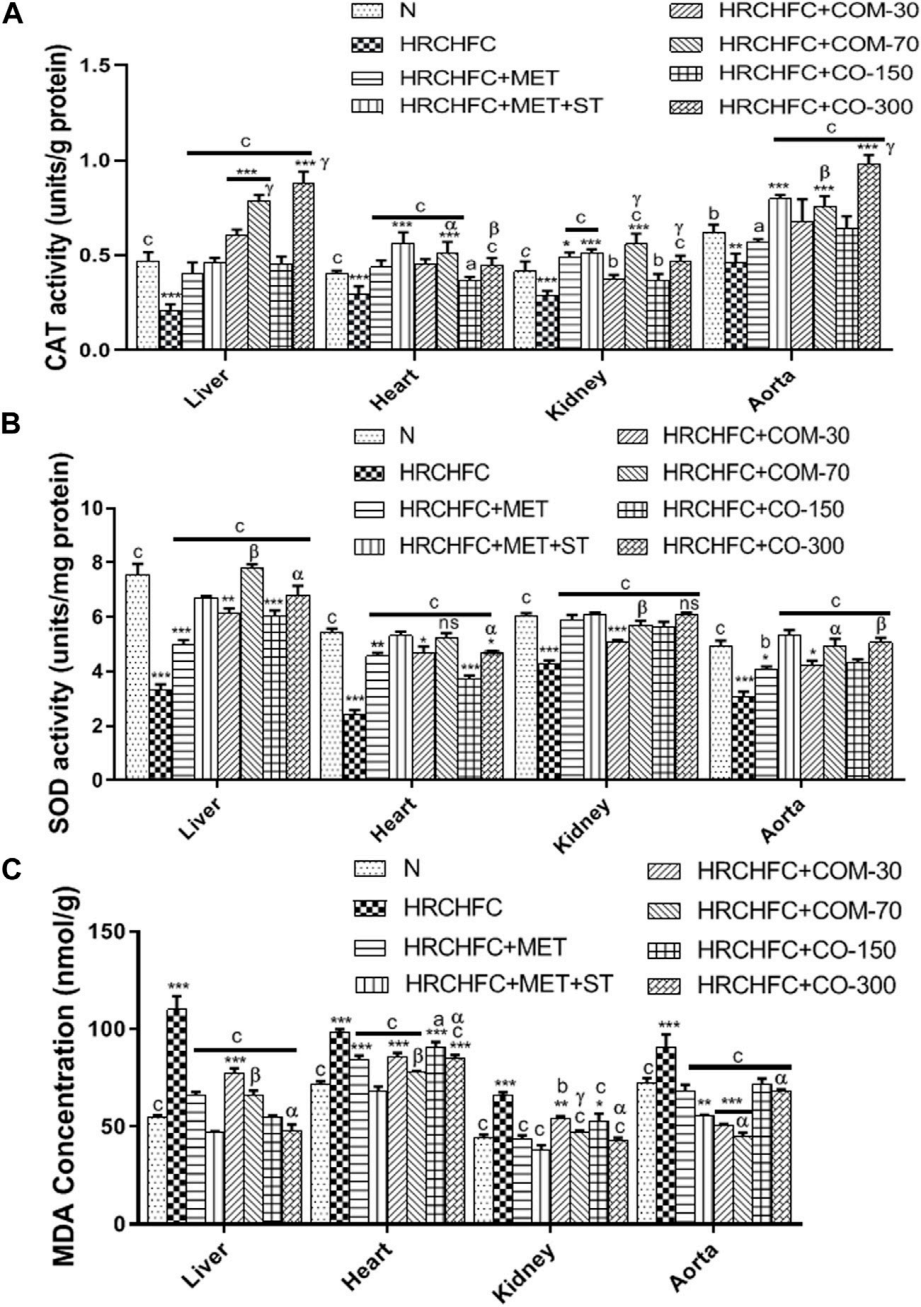
Effect of *C. odorata* and coumarin administration on **(A)** CAT, **(B)** SOD, and **(C)** MDA enzyme in various organs of HRCHFC- diet induced CMS rats. Values are expressed as mean ± SEM (*n* = 6), where ***p < 0.001, **p < 0.01, *p < 0.05 vs. normal control and ^c^ *p* < 0.001, ^b^ *p* < 0.01 and ^a^ *p* < 0.05 vs. CMS control group using one-way ANOVA followed by Dunnett’s test. ns = non-significant, ^γ^ *p* < 0.001, ^β^ *p* < 0.01, ^α^ *p* < 0.05 shows effect of low vs. high dose of *C. odorata* and coumarin (Student’s t-test).

Figure 8 shows the effect of *C. odorata* and coumarin supplementation on oxidative stress markers in liver, heart, kidney, and aorta tissue homogenates. *C. odorata* and coumarin-treated rats significantly (*p* < 0.001) raised catalase and superoxide dismutase enzymes in their livers, heart, kidneys, and aortas, which were found depleted in the organs of HRCHFC-fed rats. The detrimental effect of lipid peroxidation in HRCHFC-fed rats markedly (*p* < 0.001) decreased, representing an attenuation in MDA levels when treated with *C. odorata* and coumarin.

### Effect on histopathology of organs

**FIGURE 9:**
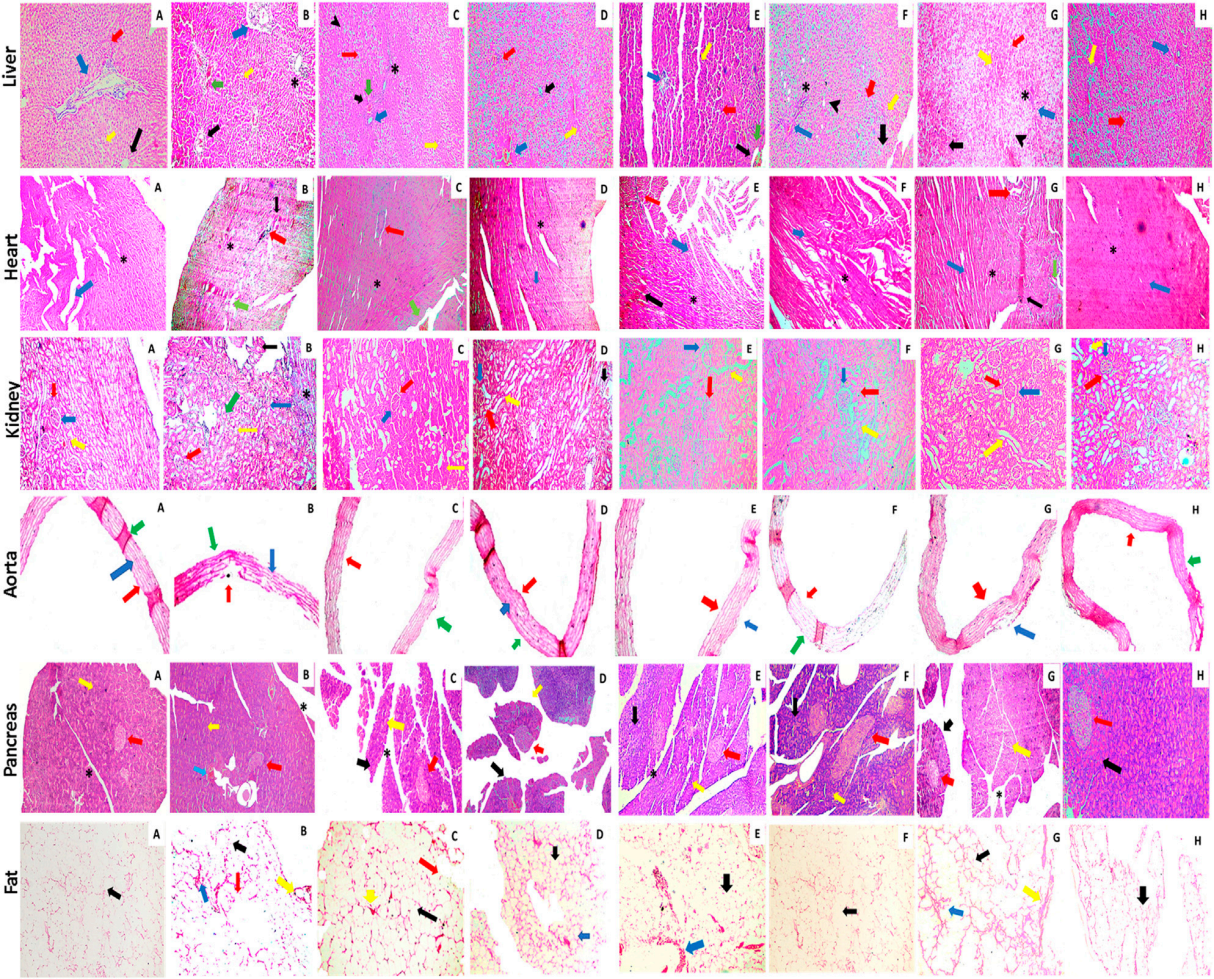
Photomicrographic illustration of liver, heart, kidney, aorta, and fat tissue sections stained with H&E dye showing effect of administration of *C. odorata*: aqueous methanolic extract of *C. odorata* (150 and 300 mg/kg) and coumarin: (30 and 70 mg/kg) in HRCHFC diet-induced CMS and normal rats. Liver: black, yellow, green, red, blue arrows, stearic, and arrow head, respectively, show central vein, sinusoids, necrosis, hepatocyte, branch of portal triad, cell infiltration, and fat collection. Heart: black, green, red, blue arrows, and stearic, respectively, show inflammation, necrosis, cell infiltration, myofibrils, and cardiomyocytes myofibrils. Kidney: red, blue, yellow, black arrows, and stearic, respectively, show Bowman’s capsule, glomerulus, distal convoluted tubules, shrinkage of cortex, and cell infiltration. Pancreas: yellow, black, blue, red arrows, and stearic, respectively, show exocrine pancreas, pancreatic acinar cells, cell infiltration, islets of Langerhans, and intra lobular duct. Aorta: red, blue, and green arrows show tunica adventitia, media, and intima layers of blood vessel, respectively. Fat: yellow, green, red, and blue arrows, respectively, show collagen fiber, adipose cell, fat deposit, and inflammation.

The histopathological examination of *C. odorata* and coumarin-treated liver tissues revealed a restored normal or intact texture of hepatocyte with a lesser appearance of fat deposition, reduced hepatic lesions, and necrosis with diminished inflammatory cells. A normal portal vein and dilation of sinusoids were observed in treated groups, while HRCHFC-fed rats caused degenerative and vacuolized hepatocytes, damaged hyaline with fat deposition, inflammatory cell infiltration, and congested sinusoids. The heart tissues of treated rats revealed non-infracted architecture or intact branches of myocardium, lesser cell infiltration, necrosis, and inflammation, and reduced edema with a restoration of myofibril integrity. Restored normal glomerulus with no hyperemia, normal basement membrane and capillaries, no shrinkage of the renal cortex, and absence of inflammatory cells was observed in the kidney tissues of treated rats, whereas the aortic tissues of treated groups presented significant progression of foam cells, repair of elastic fibers, and lessened fat deposition in the tunica media layer of the aorta. An organized pattern, normal architecture, and the regeneration and development of islets of Langerhans and β-cells were evident in the pancreatic tissue of *C. odorata* and coumarin-treated rats. The dilation of the intra-lobular duct and reduced inflammation of pancreatic tissue were also observed in treated groups. Treated rats reversed the size and number of adipocytes with less inflammation in the fat pad of the epididymal tissues (Figure 9).

## Discussion

Due to a high mortality and morbidity rate, CMS presents a serious global health risk, thus demanding effective therapeutic alternatives (Agrawal et al., 2018). The results of the present study clearly demonstrated that *C. odorata* and coumarin improved the characteristic features of CMS by attenuating obesity, dyslipidemia, oxidative stress, insulin resistance, glucose intolerance, and hypertension in HRCHFC-fed rats. A chronic administration of a HRCHFC diet with additional cholic acid induces oxidative stress, and hemodynamic and metabolic alteration (Prasatthong et al., 2021) that mitigate the cellular metabolism of dietary ingredients, resulting in the development of obesity and related comorbidities such as hyperglycemia, glucose intolerance, dyslipidemia, arterial hypertension, insulin resistance, systemic inflammation, and hepatic steatosis (Senaphan et al., 2015).

The traditional use of *C. odorata* in the treatment of diabetes and its α-glucosidase-inhibitory activity (Abbasi M. et al., 2014) provide a sound basis to its potential use as an anti-diabetic. Furthermore, *C. odorata* has high amount of coumarin as phenolic content (Joshi et al., 2019). Thus, the beneficial effects of *C. odorata* and coumarin are attributed to its increased antioxidant activity and attenuation of excessive adipocytokine production, including TNF-α, IL-6, leptin, chemerin, and adiponectin with the regulation of body weight and feed intake. A notable improvement in hepatic steatosis and dyslipidemia is strongly related to the modulation of HMG-CoA reductase and inflammatory cytokines. *C. odorata* and coumarin reversed HRCHFC- diet induced hypertension, possibly through a NO mediated pathway.

Phytochemical analysis and the oxidative potential of plant extract were examined by HPLC, TFC, TPC, and radical scavenging activities (DPPH, H_2_O_2_, reducing power). The conventional FTIR spectrum of *C. odorata* showed a narrow peak at 3,245.75 cm^–1^ (O–H and N–H), while a peak at 2,938.41 cm^–1^ identifies a C–H stretch. Sharp peaks at 2,366.59, 2059.31 cm^–1^ (C≡N, C≡C) indicate the presence of a CH_2_ group. The peaks present at 1,607.74, 1,521.24 cm^–1^ (C=O), 1,442.22 cm^–1^ (aromatic ring), 1,358.66 (C–H), and 1,245.68 (O–H) indicate that *C. odorata* contains flavonoids, alkaloids, and phenolics. Interval peaks at 1,014.86 and 926.32 cm^–1^ show a C–O–C group of saponins. The HPLC analysis revealed a quantitative amount of a variety of polyphenols, mainly phenolic acids, flavonoids, and their derivatives in aqueous methanolic extract of *C. odorata*: quercetin, catechin, gallic acid, caffeic acid, ferulic acid, chologenic acid, syringic acid, p-coumaric acid, sinapic acid, and a higher expression of coumarins. Many of these polyphenols are reported to have an ameliorating effect on obesity and other features of metabolic syndrome, including glucose intolerance, dyslipidemia, hypertension, and oxidative stress (Prince et al., 2021). Thus, the results obtained from this study that indicate the ability of *C. odorata* to inhibit obesity and associated metabolic alterations are attributed in part to the presence of these polyphenolic compounds—amongst these, coumarin contributes prominently to the overall effectiveness of *C. odorata* in CMS.

After the eighth week of induction with the HRCHFC diet, significant weight gain and food consumption were observed due to the addition of added vegetable oil (fat), fish meal (protein), and refined carbohydrate. This may intricate hyperplasia and hypertrophy of adipocytes (BrahmaNaidu et al., 2014).

As a result of high caloric diet intake, the energy metabolism is compromised in the liver (Gutierrez**-**Salmean et al., 2014). Low energy levels stimulate the hunger center, increasing food intake and resulting in the development of CMS in animals. This suggests that low hepatic energy influences feeding behavior and ultimately causes an increase in body weight (Zhang et al., 2021). This study’s results clearly showed that weight gain is consistent with feed intake. The administration of *C. odorata* and coumarin significantly and in a dose-dependent manner reduced weight gain and feed intake during the experimental period. Treated groups probably increased energy consumption and enhanced fat oxidation, thus causing weight loss in HRCHFC-fed rats (Mabrouki et al., 2020). Organ weight increase, particularly of the liver and heart, in diseased animals might be due to inflammation, hypertrophy, and steatosis (Nwakiban**-**Atchan et al., 2022), while a marked decrease was observed in treated groups. These findings indicate the anti-obesity effect of *C. odorata* and coumarin. This was also confirmed by morphological examination of the adipocyte of fat pads, where a significant increase in the size and number of adipocytes were reversed to normal in the treatment groups.

It is known that metabolic complications related to obesity are prevented by inhibiting elevated adipokine levels. HRCHFC diet-induced obesity and related diseases like diabetes, insulin resistance, and increased fat mass are associated with the dysregulation of adipocytokines. These are secreted from adipocyte, in which adiponectin has anti-diabetic, anti-atherogenic, and anti-inflammatory properties (Sasso et al., 2019). Leptin is appetite regulator hormone which plays a major role in balanced feed intake and body weight control. Leptin secretion is positively correlated with the degree of triglycerides stored in adipose tissues (BrahmaNaidu et al., 2014). Elevated chemerin levels show the interlinking of metabolic syndrome and obesity by producing pathophysiological complications such as increased fat, glucose, lipid metabolism, inflammation, and elevated blood pressure that lead to hyperlipidemia and insulin resistance. Chemerin also stimulates insulin-mediated glucose uptake and improves insulin action in 3T3-L1 adipocyte to treat obesity and insulin resistance (Takahashi et al., 2008). The present study highlights *C. odorata* and coumarin treated rats as presenting high levels of adiponectin and reduced levels of leptin and chemerin. Reduced leptin may be due to decreased body fat mass and increased leptin sensitivity (BrahmaNaidu et al., 2014). These observations indicate that the regulation of adipokines in treated groups may be due to reduced lipid buildup in adipocytes, which is also consistent with the findings on the lipid profile in treated rats.

Insulin resistance is considered a hallmark for metabolic syndrome and diabetes (Senaphan et al., 2015). It causes elevated adipokine secretion, which develops hyperglycemia or glucose intolerance for metabolic adaptation. A HRCHFC diet produces hepatic insulin resistance, possibly by elevating the circulation of free fatty acid (FFA) levels (Prince et al., 2021). Elevated FFA in HRCHFC-fed rats leads to increased serum glucose levels that manifest obesity-induced glucose intolerance and inhibit muscle glucose uptake (Lasker et al., 2019). Excessive FFA levels also damage the pancreas to produce insulin to combat hyperglycemia (BrahmaNaidu et al., 2014). *C. odorata* and coumarin alleviate hyperglycemia by reducing FBG levels to prevent insulin resistance. The observed effects might be achieved due to the presence of a diverse nature of phytoconstituents in the plant. This effect is in line with previously reported *in vitro* α-glucosidase activity of *C. odorata* (Abbasi M. et al., 2014), which also supports the anti-diabetic action of *C. odorata*. *C. odorata* and coumarin administration caused an improvement in HRCHFC diet-induced impaired glucose tolerance in a dose-dependent manner, evident by its positive impact in the oral glucose tolerance test and assessed FBG levels. Treatment groups markedly reduced their blood glucose levels following by insulin administration, thus showing an improvement in insulin sensitivity. This effect possibly occurs due to improvement in one or more defects *viz.* insulin receptors, insulin receptor substrate, glucose transporters, or glycosylated enzymes. The liberation of inflammatory cytokines is also a strong inducer of insulin resistance (Senaphan et al., 2015). Improved insulin resistance was also shown in the pancreatic tissue of *C. odorata* and coumarin treated rats, where damaged islets of Langerhans, atrophic β-cell, reduced β-cell mass, and congested intra-lobular duct were revived to normalcy.

Atherogenic dyslipidemia is an intricate disorder related to obesity, diabetes, and metabolic syndrome that enhances the progression of CVD risk (Prince et al., 2021). HRCHFC diet-induced hypertriglyceridemia causes endothelial dysfunction, atherosclerosis, and increased oxidative stress which can be prevented by antioxidants by altering the lipid metabolism (Aziz et al., 2013). In our study, a HRCHFC diet with cholesterol and cholic acid increased TG and TC levels while decreasing HDL-C levels, possibly by increasing FFA in the liver due to insulin deficiency and/or resistance. Hence, more TGs are absorbed into adipose tissues (Nwakiban-Atchan et al., 2022). The reduced liberation of FFA results in elevated levels of VLDL (Ojetola et al., 2021). These changes were endorsed by the observed decreased HMG-CoA reductase activity (Nepal et al., 2011) and TG uptake by peripheral tissues (Sharma et al., 2012). However, reduced HDL-C levels are due to activated lipoprotein lipase and lecithin cholesterol-acyl transferase (LCAT) with reduced cholesterol catabolism (BrahmaNaidu et al., 2014)*.* Treatment with *C. odorata* and coumarin noticeably improved the lipid profile by causing a decrease in HMG-CoA reductase activity, and TG and TC levels, while increasing HDL-C levels—possibly by inhibiting lipid synthesis—and reducing cholesterol absorption and secretion from the intestine. HMGR (3-hydroxy-3-methylglutaryl-coenzyme A reductase) limits the endogenous enzyme for cholesterol synthesis and is also used to catalyze HMG-CoA reductase conversion to mevalonate (BrahmaNaidu et al., 2014). HMG-CoA reductase inhibitors (lipid-lowering agents) are used as first line agents in treating hyperlipidemia (Javaid et al., 2021). Our study also showed increased HMGR levels in HRCHFC-fed rats, which is an established fact of obesity related to CMS (Kalaivani et al., 2019). Most therapeutic agents that decrease TC levels may also potentially block HMGR enzymes (Istvan, 2002). These findings suggest that *C. odorata* and coumarin possess anti-dyslipidemic effects.

The elevated hepatic enzymes released in HRCHFC-fed rats may be due to the modified plasma membrane permeability of hepatocytes and biliary obstruction, thus increasing the risk of developing non-alcoholic fatty liver disease (NASH) (Suman et al., 2016). Hepatocyte damage followed by oxidative damage leads to liver injury. Furthermore, higher oxidative stress, dysregulated adipocytokine production, and mitochondrial dysfunction are causative factors of NASH (Sharma et al., 2012). Administration of *C. odorata* and coumarin to rats caused a remarkable dose-dependent reduction in serum ALT and AST levels to relieve hepatic steatosis through protecting hepatic dysfunction. Increased urea and creatinine levels in CMS-developed animals were found to be strongly associated with renal damage (Bamanikar et al., 2016). Treatment with *C. odorata* and coumarin offered a significant attenuation in serum urea and creatinine levels to prevent nephropathy. Damaged renal tissues in HRCHFC-fed rats were produced by ROS production (El-Alfy et al., 2005). Intake of *C. odorata* and coumarin in CMS animals restored renal architecture in HRCHFC-fed rats, possibly by neutralizing free radicals in these tissues. Our results are in accordance with previously published data (Bhatti et al., 2022). The observed findings might be attributed to the presence of abundant phytochemicals in *C. odorata*, their antioxidant potential, and anti-inflammatory properties.

Manifested features of hepatic steatosis were found to be reduced in hepatic tissues of treated groups, which was evident in revived hepatocyte texture, reduced vacuolization, and congestion in the central vein as well as minimized fat deposition. Similarly, the kidney tissues of treated groups also revived congested glomerular blood vessels, necrotic tubules, inflammation, and cloudy deteriorative parts to a more normal architecture.

The administration of HRCHFC diet for 8 weeks initiated the development of obesity and low-grade chronic inflammation mediated by the liberation of pro-inflammatory cytokines like IL-6 and TNF-α (Sasso et al., 2019). The literature also showed that elevated inflammatory biomarkers and free radicals cause insulin resistance commonly associated with endothelial dysfunction, dyslipidemia, and hyperglycemia (Senaphan et al., 2015). Elevated FFAs in HRCHFC-fed rats directly activate the macrophages to secret pro-inflammatory cytokines (TNF-α primarily stimulate others) that render insulin resistant in peripheral tissues (Jung et al., 2011). This study showed that treatment with *C. odorata* and coumarin exhibited a significant dose-dependent decrease in serum TNF-α and IL-6 levels. Thus, increased antioxidant enzyme activity and the suppression of inflammatory markers show potential in treating hypertension, insulin resistance, diabetes, obesity, and hepatic steatosis.

*C. odorata* and coumarin-treated groups displayed significantly elevated catalase and superoxide dismutase enzyme levels compared to HRCHFC-fed rats. SOD and CAT are considered first-line defense anti-oxidant enzymes against ROS (Nepal et al., 2011). The upregulation of CAT levels prevents atherosclerosis and blocks angiotensin-II mediated aortic wall hypertrophy. Elevated SOD levels prevent the inactivation of NO and protect the liver from oxidative damage by scavenging molecular oxygen (Sharma et al., 2012). Thus, the reduction offered in CAT and SOD levels might demonstrate the cardio and hepatoprotective effects of *C. odorata* and coumarin. A HRCHFC diet administered to animals causes oxidative stress which is either enzymatically or non-enzymatically reflected as higher level of TBARS or reduced levels of SOD, CAT, GPx, GSTs, and GSH (Aziz et al., 2013). Earlier studies reported that increased oxidative stress in HRCHFC-fed rats is usually an outcome of inflammation, insulin resistance, hypertension, dysregulated adipocytokines, and NASH (Kabelova et al., 2021). Oxidative stress develops with increased ROS and reduced antioxidant enzymes, resulting in excessive molecular oxygen and hydrogen peroxide that lead to initiate lipid peroxidation (Sharma et al., 2012). *C. odorata* and coumarin administration attenuated lipid peroxidation levels, which was evident by reduced MDA levels in treated rats, and thus offers protection against hypertension. Theses finding on *C. odorata* and coumarin may highlight their therapeutic potential in treating CMS.

Hypertension is a characteristic feature of metabolic syndrome related to obesity (Suman et al., 2016). HRCHFC-fed rats show multiple underlying mechanism(s) that mediate hypertension, including endothelium dysfunction, oxidative degradation of NO, and diminished eNOS activity (Ghibu et al., 2019). Previous studies have reported that decreased NO production in vascular endothelium accounts for impaired vascular function that can result in vascular diseases including hypertension. Endothelial cells also form active oxygen radicals in response to hyperlipidemia and inflammation, which causes destruction of NO and vasoconstriction. A growing body of evidence suggests that HRCHFC diet leads to increased levels of LDL-c and oxidative stress, which was also observed in our study. This increases the levels of oxidized LDL which has a suppressive action on endothelial NO synthase. ROS also increases cytosolic Ca^2+^ in smooth muscle as well as sensitizing the muscle contractile apparatus in vascular smooth muscle, further augmenting vasoconstriction (Roberts et al., 2000). Elevated ROS, mainly superoxide anions, can strongly inactivate NO, leading to the development of vascular damage (Sharma et al., 2012). Moreover, superoxide anions fuses to NO and tyrosine to make peroxynitrite and nitrotyrosine, respectively, which ultimately attack proteins, lipids, and DNA to cause cellular damage. It has also been reported that hyperlipidemia, insulin resistance, and inflammatory contributors in CMS have repressive effects on eNOS. These increase the wall thickness of conduit vessels, leading to deceased NO synthesis for the development of hypertension (Ojetola et al., 2021). Vasomotor tone modulation and vascular remodeling can also be managed by rendering oxidative stress biomarkers, which are known to mediate cardiovascular complications (Senaphan et al., 2015). Several studies have shown that the intake of natural agents enriched with antioxidants has proven beneficial effects against oxidative stress-augmented pathophysiological anomalies. (Nepal et al., 2011). In our study, the observed anti-hypertensive effect on the part of *C. odorata* and coumarin by reducing systolic blood pressure (SBP), diastolic blood pressure (DBP), mean blood pressure (MBP), and heart rate (HR) might be associated with its anti-oxidant, antihyperlipidemic, and/or anti-inflammatory activities.

Furthermore, treated rats showed a marked improvement in the morphological features of aortic and cardiac tissues by exhibiting restored myocardial fiber texture, reduced inflamed necrotic areas, and a repaired tunica media layer with less fat deposition, indicating the cardiovascular beneficial potential of the test materials.

Many studies have shown that the phytoconstituents of *C. odorata* account for its multiple therapeutic properties (Shahzadi et al., 2012; Abbasi M. et al., 2014; Abbasi M. A. et al., 2014; Abbasi et al., 2017; Joshi et al., 2019). Flavonoids and phenolic compounds have much biological potential due to their antioxidant and free radical scavenging properties (Prince et al., 2021). Furthermore, it is unclear which constituent is exactly responsible for attenuating the characteristic features of CMS. Hence, further investigation is needed to determine the biologically active constituent responsible for the actions of *C. odorata* against CMS.

## Conclusion

*C. odorata* and coumarin improved obesity and dyslipidemia through the modulation of adipocytokines (leptin, adiponectin, chemerin), inhibition of HMG-CoA reductase, and attenuation of the lipid profile. Treatment with *C. odorata* and coumarin offered an anti-hypertensive effect and caused modulation in insulin resistance, possibly through its effect on amended oxidative stress biomarkers (SOD, CAT, and MDA), inflammatory mediators (TNF-α and IL-6) and improved insulin sensitivity. Its protective effect for hepatic steatosis was evident by its positive influence on hepatic function markers (LFTs). Thus, this study highlights the therapeutic potential of *C. odorata* and coumarin for treating cardiometabolic syndrome.

## Data Availability

The original contributions presented in the study are included in the article/Supplementary Material; further inquiries can be directed to the corresponding author.

## Abbreviations

ALT, alanine transaminase; AST, aspartate transaminase; CAT, catalase; CMS, cardiometabolic syndrome; CRP, c reactive protein; CVD, cardiovascular diseases; DBP, diastolic blood pressure; ELISA, enzyme-linked immunosorbent assay; HDL, high density lipoprotein; HMG-CoA reductase, (3-hydroxy-3-methyl-glutaryl-coenzyme A) reductase; HR, heart rate; HRCHFC, high refined-carbohydrate-high fat-cholesterol; IL-6, interlukin-6; IR, insulin resistance; LFTs, liver function tests; MBP, mean blood pressure; MDA, Malondialdehyde; NIBP, non-invasive blood pressure; RFTs, renal function tests; ROS, reactive oxygen species; SBP, systolic blood pressure; SOD, superoxide dismutase; TC, total cholesterol; TG, total triglycerides; TNF-α, tumor necrosis factor.
